# High-resolution genome-wide DNA methylation maps of mouse primary female dermal fibroblasts and keratinocytes

**DOI:** 10.1186/1756-8935-7-35

**Published:** 2014-12-02

**Authors:** Raghunath Chatterjee, Ximiao He, Di Huang, Peter FitzGerald, Andrew Smith, Charles Vinson

**Affiliations:** Laboratory of Metabolism, National Cancer Institute, National Institutes of Health, 37 Convent Drive, Bethesda, MD 20892 USA; Human Genetics Unit, Indian Statistical Institute, 203 B. T. Road, Kolkata, 700108 India; NCBI, National Institutes of Health, 8600 Rockville Pike, Bethesda, MD 20894 USA; Genome Analysis Unit, Genetics Branch, National Cancer Institute, National Institutes of Health, 37 Convent Drive, Bethesda, MD 20892 USA; Molecular and Computational Biology, University of Southern California, 1050 Childs Way, Los Angeles, CA 90089 USA

**Keywords:** CG methylation, Hypomethylated regions, HMR, Methylome, CTCF, C/EBPβ, Keratinocytes, Fibroblasts

## Abstract

**Background:**

Genome-wide DNA methylation at a single nucleotide resolution in different primary cells of the mammalian genome helps to determine the characteristics and functions of tissue-specific hypomethylated regions (TS-HMRs). We determined genome-wide cytosine methylation maps at 91X and 36X coverage of newborn female mouse primary dermal fibroblasts and keratinocytes and compared with mRNA-seq gene expression data.

**Results:**

These high coverage methylation maps were used to identify HMRs in both cell types. A total of 2.91% of the genome are in keratinocyte HMRs, and 2.15% of the genome are in fibroblast HMRs with 1.75% being common. Half of the TS-HMRs are extensions of common HMRs, and the remaining are unique TS-HMRs. Four levels of CG methylation are observed: 1) total unmethylation for CG dinucleotides in HMRs in CGIs that are active in all tissues; 2) 10% to 40% methylation for TS-HMRs; 3) 60% methylation for TS-HMRs in cells types where they are not in HMRs; and 4) 70% methylation for the nonfunctioning part of the genome. SINE elements are depleted inside the TS-HMRs, while highly enriched in the surrounding regions. Hypomethylation at the last exon shows gene repression, while demethylation toward the gene body positively correlates with gene expression. The overlapping HMRs have a more complex relationship with gene expression. The common HMRs and TS-HMRs are each enriched for distinct Transcription Factor Binding Sites (TFBS). C/EBPβ binds to methylated regions outside of HMRs while CTCF prefers to bind in HMRs, highlighting these two parts of the genome and their potential interactions.

**Conclusions:**

Keratinocytes and fibroblasts are of epithelial and mesenchymal origin. High-resolution methylation maps in these two cell types can be used as reference methylomes for analyzing epigenetic mechanisms in several diseases including cancer.

Please see related article at the following link: http://www.epigeneticsandchromatin.com/content/7/1/34

**Electronic supplementary material:**

The online version of this article (doi:10.1186/1756-8935-7-35) contains supplementary material, which is available to authorized users.

## Background

Most DNA modification in mammals is the methylation of cytosine (5mC) in the context of the CG dinucleotide [1–3]. Non-CG cytosine methylation is observed in plants, human embryonic stem cells and neuronal cells [4, 5]. CG dinucleotides are underrepresented in the mammalian genome, a presumed consequence of the spontaneous deamination of the methylated cytosines to thymine [6]. A total of 5% of the CG dinucleotides in the mammalian genome occur in approximately 20,000 clusters termed CG Islands (CGIs), with approximately half being at the promoters of housekeeping genes [7]. CGI associated promoters tend to be unmethylated irrespective of their gene expression, while CG-poor promoters tend to be methylated and are associated with tissue specific genes [8]. The present understanding of CG methylation suggests a diverse role in genome regulation including in the determination of cell type specificity, cellular differentiation, suppression of transposable elements, X-chromosome inactivation, genomic imprinting, DNA-protein interaction and tumerogenesis [9–17]. Tissue-specific hypomethylated regions (TS-HMRs) have previously been identified and are associated with the tissue-specific gene expression in human and mouse cells [15, 18, 19].

Recently, several single nucleotide resolution maps of DNA methylation in human [5, 20], mouse [19], plants [4] and honey bee [21] have been possible because of long read-lengths of the high-throughput Illumina sequencing platform. These studies revealed several interesting observations: hypermethylation of CGI shores toward the gene body was shown to be positively correlated with gene expression [18, 22]; expressed protein-coding genes appear to have high CG methylation over their gene body [15, 18, 23, 24]; CG methylation undergoes dynamic changes at the regulatory regions outside the core promoter during cellular differentiation [14]. The hypermethylated regions at the edges of CGIs towards the gene body [18, 22] potentially represent methylated exons that are associated with increased gene expression [15, 18, 23, 24]. CG methylation increases sequence-specific binding of some transcription factors, which is essential for gene activation, particularly in methylated tissue-specific promoters [8, 12, 15, 25]. However, the characteristics and dynamic nature of tissue-specific DNA methylation changes remain an open problem. Genome-wide cytosine methylation profiling at a single nucleotide resolution in different primary cells of the mammalian genome could help to unravel the characteristics and functional prediction of the TS-HMRs.

Changes in CG methylation occur throughout development and pathology. A major event during developmental differentiation in mammals is the demethylation of regions of DNA to produce TS-HMRs that may function to activate expression of nearby tissue-specific genes [14, 15, 18, 25–28]. Single base-pair resolution maps of cytosine methylation have been published for mammals reinforcing the idea that CG demethylation occurs in clusters, typically in regions of high CG density [5, 18–20, 29]. A recent examination of 42 methylomes from human cells and tissues identified changes in methylation in 22% of the approximately 20 million CG dinucleotides in the genome [28].

Here, we compared the DNA methylation maps in newborn female mouse primary dermal fibroblasts and keratinocytes derived from skin. Understanding the epigenomic fingerprint of these two cell types may shed light on the epithelial to mesenchymal transition (EMT) observed in cancer [30]. Primary cultures, in comparison to cell lines have several advantages. Previous studies have shown that cells with increasing passage number lead to aberrant epigenetic changes [14]. To help maintain the proper epigenetic state of these cells, we cultured primary keratinocytes for three days and dermal fibroblasts for eight days without passage. We determined the high-resolution genome-wide DNA methylation maps of these two primary cultures using bisulfite-sequencing and compared these maps to gene expression profiles obtained using high-throughput RNA-sequencing, to evaluate the role of CG methylation in cell type specificity determination.

## Results

### Genome-wide CG methylation in mouse primary keratinocytes and dermal fibroblasts

**Figure 1:**
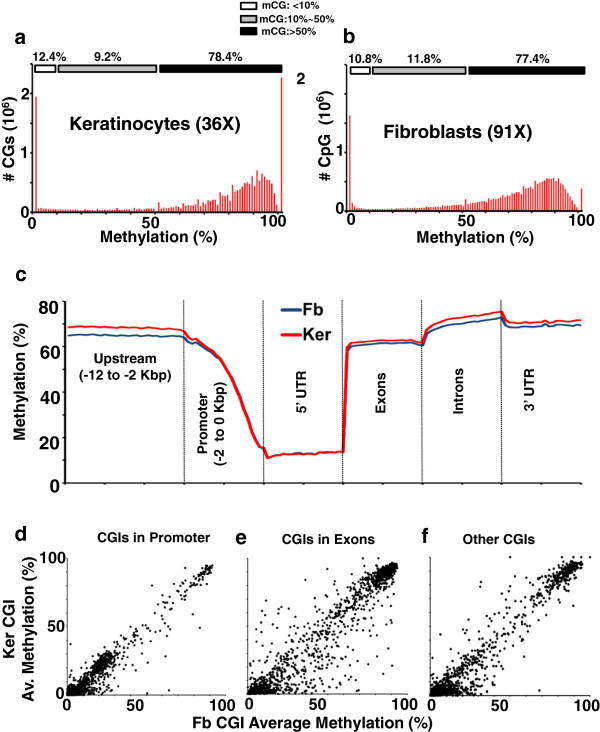
**CG Methylation in keratinocytes and fibroblasts. (a-b)** Methylation status of all CGs in **(a)** keratinocytes (Ker) at 36X coverage and in **(b)** fibroblasts (Fb) at 91X coverage. **(c)** Average methylation of CGs in different genomic locations. Each CG is grouped one of the six genomic locations (Upstream, Promoter, 5’ UTR, Exon, Intron, or 3’ UTR) according to the transcription start site (TSS) and coding sequence (CDS). **(d-f)** Comparison of average CGI methylation for Fb versus Ker in different CGI groups: **(d)** CGIs in Promoter, **(e)** CGIs in Exons, and **(f)** the other CGIs.

Single base pair resolution cytosine methylation maps of newborn female mouse primary dermal fibroblasts [31] and epidermal keratinocytes were generated using bisulfite conversion of genomic DNA and next-generation based high-throughput Illumina sequencing technology. We generated 3.75 [31] and 1.38 billion paired-end 102-bp reads for dermal fibroblasts and keratinocytes, respectively [see Additional file 1: Table S1a-b]. We aligned 67.8% and 68.1% of the reads uniquely to the reference mouse genome (mm9) with a false discover rate of 0.007% and 0.004% and generated an average read depth of 91X for primary dermal fibroblasts [31] and 36X for keratinocytes [see Additional file 1: Table S1c]. Of the 21,342,779 CGs in the haploid mouse genome; 13,132,502 CGs are in unique regions; of these, 98.3% in fibroblasts and 98.2% in keratinocytes are covered by at least one read [see Additional file 2: Figure S1a-d, Additional file 1: Table S1d]. To compare the two methylomes, we used a subset of the dermal fibroblast data that is comparable to the keratinocyte methylome coverage [see Additional file 2: Figure S1e-f, Additional file 1: Table S1c]. The average cytosine methylation of CG dinucleotides in fibroblasts and keratinocytes is 65% and 67% respectively [see Additional file 1: Table S1c]. We observed 5mC in non-CG methylation of approximately 0.06% in both primary cells, which represents both CHG and CHH methylation, and we did not include that in further analysis [see Additional file 2: Figure S1g-h, Additional file 1: Table S2a]. Keratinocytes have a higher number of both completely unmethylated and completely methylated CGs in comparison to the dermal fibroblasts (Figure 1a-b, [see Additional file 1: Table S2b]), which may reflect the more differentiated state of the keratinocytes. Approximately 2.5 million CGs were sparsely methylated (<10% methylated), approximately 1.8 million CGs were low-methylated (10% to 50% methylated), and the remaining approximately 15.5 million CGs were highly methylated (>50% methylated) in primary keratinocytes (Figure 1a, [see Additional file 1: Table S2c-e]), with similar results for the fibroblasts (Figure 1b, [see Additional file 1: Table S2c-e]). Adjacent CGs have a similar methylation status (Pearson’s Correlation = 0.9) but the correlation drops to 0.3 at approximately 150 bps in both primary cells, revealing the clustered nature of CG methylation [see Additional file 2: Figure S2]. Periodicity of correlation for the methylation of neighboring CG dinucleotides was also observed previously in the Arabidopsis and honey bee genomes [4, 21, 32].

The average methylation at the promoter (-2 to 0 kbps from the transcription start site (TSS)), upstream region (-12 to -2 kbps from TSS), 5’ UTR, 3’ UTR, exons and introns is similar to previous reports [4, 5, 19, 21, 24, 33]; these regulatory regions are unmethylated except for the exons. DNA methylation for keratinocytes is a little higher (approximately 4 to 5%) than for dermal fibroblasts in regions away from promoters (Figure 1c). A comparison of average methylation of different repeat elements in the two cell types showed higher methylation of long interspersed element (LINE), short interspersed element (SINE) and long terminal repeat (LTR) elements in keratinocytes than dermal fibroblasts [see Additional file 2: Figure S3, Additional file 1: Table S3]. In contrast, repetitive tRNA, rRNA and regions of low complexity showed identical methylation patterns in the two cell types [see Additional file 2: Figure S3]. CG islands (CGIs) that overlap promoters are primarily unmethylated with little difference between the two cell types (Figure 1d). In contrast, CGIs that overlap exons are more variable (*P* < .001, F-, *T*-test) (Figure 1d-f). However, gene ontology analysis of the exons that are differentially methylated in the two cells does not show any significant enrichment of any GO category. Evolutionary conservation using PhyloP analysis [34] of the CG dinucleotide in promoters and introns shows that some CG dinucleotides that are methylated are not conserved [see Additional file 2: Figure S4]. In contrast, some methylated CGs in exons are more conserved than the unmethylated CGs [see Additional file 2: Figure S4].

### Comparing hypomethylated regions in keratinocyte and fibroblast

**Figure 2:**
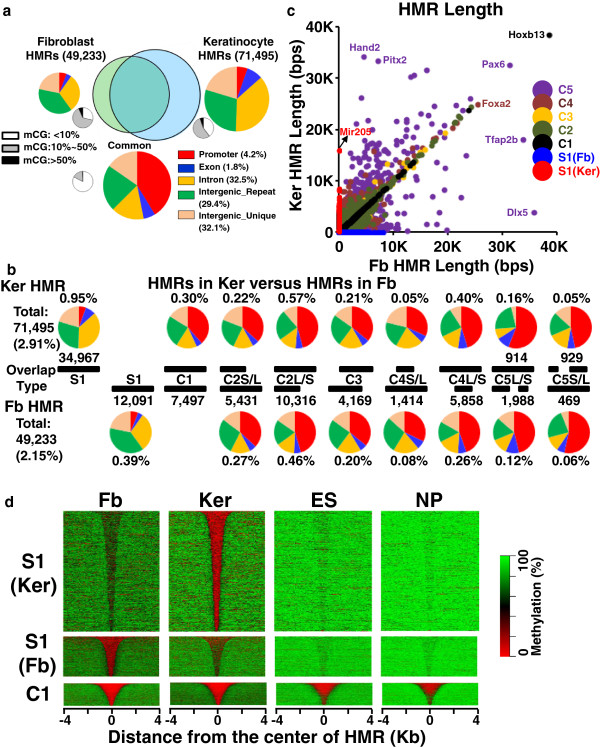
**Comparison of hypomethylated regions (HMRs) in fibroblasts and keratinocytes.** **(a)** Venn diagram showing overlapping fibroblast (91X) and keratinocyte (36X) HMRs. Big color pie charts show genomic localization (five groups) of tissue-specific and common HMRs. The common HMRs are enriched in the promoter region (red). Small black-white pie charts show the composition of CG methylation. Low-methylated CGs (10% to 50% methylated, in gray) are enriched in tissue-specific HMRs while unmethylated CGs (<10% methylated, in white) are enriched in common HMRs. **(b)** Overlap between keratinocytes (Ker) and fibroblast (Fb) HMRs. The percent of the genome represented for two tissue-specific (S1) and eight groups of overlapping HMRs (C1-C5) are shown. Pie charts show fraction of HMRs in five genomic regions. **(c)** Length comparison between keratinocytes and fibroblasts HMRs. For the tissue-specific HMRs (S1, blue for Fb, and red for Ker), the length of HMRs in the other cell is 0. **(d)** Heat map of CG methylation for the tissue-specific and common HMRs along with two published methylomes for embryonic stem (ES) and neuronal progenitor (NP) cells [19]. The common HMRs are unmethylated (red) in all four methylomes. Fibroblast and keratinocyte specific differentially methylated regions (S1) are more methylated than the common HMRs (C1). Keratinocyte specific HMRs in fibroblasts show intermediate methylation compared to neighboring sequences and vice-versa.

A two-state Hidden Markov Model (HMM)-based method was used to detect hypomethylated regions (HMRs) [18] in both methylomes [see Figure 2, Additional file 2: Figure S5a-b, Additional file 1: Table S2c-e]. We compared the fibroblast [31] and keratinocyte HMRs to identify overlapping and tissue-specific HMRs. A Venn diagram of keratinocyte HMRs (2.91% of the genome) and fibroblast HMRs (2.15% of the genome) shows 34,967 HMRs (0.95% of the genome) are only in keratinocytes and 12,091 (0.39% of the genome) are only in fibroblasts (Figure 2a-b). The remaining HMRs overlap, representing 1.96% of the genome in keratinocytes compared to 1.76% in fibroblasts and indicating that the overlapping HMRs are longer in keratinocytes (Figure 2a-b).

HMRs that overlap between keratinocytes and fibroblasts were placed into eight groups (Figure 2b, [see Additional file 2: Figure S6]). The first group (C1) has identical boundaries on both sides of the HMRs (0.30% of genome). The second group (C2) has one common boundary at one end of the HMR but are longer either in keratinocytes (0.79% of the genome) or in fibroblasts (0.73%) [22]. On average, these extended HMRs (C2) are about 280-bps long and contain 5 CGs. The remaining three groups (C3 to C5) have more complex overlapping properties as shown in Figure 2b (keratinocytes (0.87%) and fibroblasts (0.73%)). The overlapping HMRs include 84% of all CGIs [see Additional file 1: Table S4, Additional file 2: Figure S6] [7, 35, 36]. All eight overlapping HMR groups are mostly enriched in promoters (Figure 2a-b, [see Additional file 2: Figure S6]) and in contrary, tissue-specific HMRs are enriched mostly in introns and intergenic regions (Figure 2a-b, [see Additional file 2: Figure S6]) [14, 19].

Figure 2c presents the length of HMRs in keratinocytes and fibroblasts (Figure 2c, [see Additional file 2: Figure S5a-b]). The *Hoxb13* locus is the longest HMR (38,568 bps) in keratinocytes and fibroblasts with identical boundaries in both (Figure 2c, [see Additional file 2: Figure S5c]). For the keratinocyte-specific HMRs, the average length is 722 bps with 11 CG dinucleotides, and the longest is approximately 16,000 bps covering the *Mir205* locus (Figure 2c, [see Additional file 2: Figure S5b,d]) whereas for fibroblast-specific HMRs, the average length is 862 bps with ten CG dinucleotides, and the longest is 8,100 bps, which is located in the *Skint6* locus (Figure 2c, [see Additional file 2: Figure S5a,e]). HMRs that overlap in the two methylomes are primarily composed of CGs that are totally unmethylated, while the tissue-specific HMRs are primarily composed of low-methylated CGs as described previously (Figure 2a, [see Additional file 2: Figure S6, Additional file 1: Table S2c-d]) [19]. This trend is also observed in the C2 class of HMRs. The overlapping part of the HMR is composed of unmethylated CGs while the extended part of the HMR is composed of CGs that are low methylated (approximately 10 to 20%) [see Additional file 2: Figure S6].

A heat map of CG methylation in common and TS-HMRs identified four levels of CG methylation: 1) total unmethylation, which is for CG dinucleotides in HMRs in CGIs that are active in all tissues; 2) 10% to 40% methylation for fibroblast and keratinocyte TS-HMRs; 3) 60% methylation for fibroblast-specific HMRs in keratinocytes, and keratinocyte-specific HMRs in fibroblast; and 4) 70% methylation for the non-functioning part of the genome (Figure 2d, [see Additional file 2: Figure S6-7]). The decrease in methylation of keratinocyte HMRs in fibroblasts is not the result of cross contamination of the cultures. This will be shown when we examine the CpG methylation [see Additional file 1: Table S2b] and the mRNA-seq data that show tissue specific gene expression. The sequences corresponding to fibroblast and keratinocyte-specific HMRs are partially demethylated in mouse embryonic stem (ES) and neuronal progenitor (NP) cells [19] (Figure 2d, [see Additional file 2: Figure S7-8]), suggesting this to be a general property of these HMRs. The C1 class of HMRs is also unmethylated in both ES and NP cells, highlighting their housekeeping functions (Figure 2d, [see Additional file 2: Figure S7]).

The common and TS-HMRs for both dermal fibroblasts and keratinocytes show higher CG density than the surrounding regions [see Additional file 2: Figure S9a-e]. SINE elements are significantly depleted inside the TS-HMRs, while in the surrounding regions they are highly enriched in comparison to randomly selected regions of the genome [see Additional file 2: Figure S10, Additional file 1: Table S3] [37, 38]. In contrast, both LTR and LINE elements are significantly depleted inside and in the surrounding regions.

### Hypomethylated regions and gene expression

We next determined the mRNA expression of dermal fibroblasts and keratinocytes using Illumina high-throughput RNA-sequencing and compared this to the HMRs. Biological replicates of the RNA-seq data for both cell types are reproducible (r >0.99) [see Additional file 2: Figure S11a-c]. Genes uniquely expressed in dermal fibroblasts showed enrichment for extracellular matrix organization, immune response and wound responsive genes, which are frequently expressed in cancer cells [see Additional file 2: Figure S11d-f, Additional file 1: Table S5a]. Keratinocyte-specific gene expression showed enrichment for keratinization and epidermis development [see Additional file 2: Figure S11d,e; Additional file 1: Table S5b].

**Figure 3:**
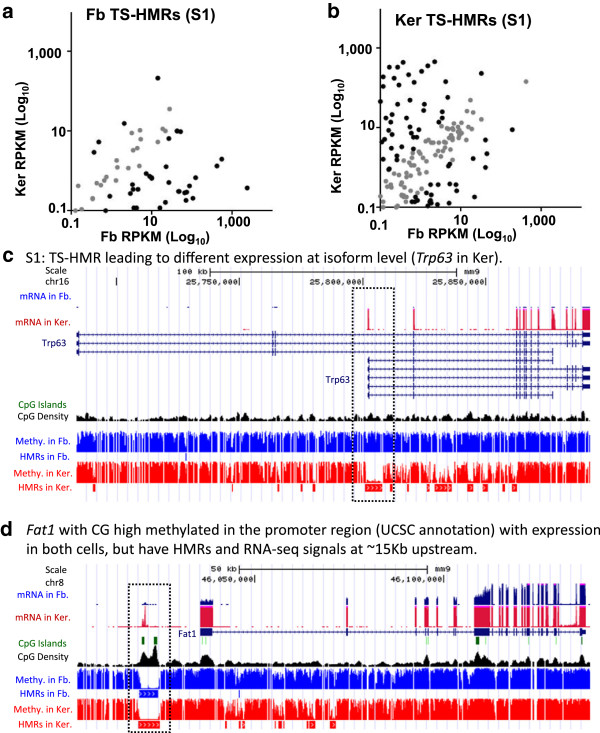
**Comparison of gene expression in fibroblasts and keratinocytes. (a-b)** Scatter plot of dermal fibroblasts and keratinocytes RNA-seq mRNA expression (reads per kilobase per million mapped reads, RPKM) for **(a)** fibroblasts specific S1 class and **(b)** keratinocyte specific S1 class of hypomethylated regions (HMRs) that have TSS within 1-kbp. **(c-d)** UCSC genome browser screen shots as examples for genes in different group of HMRs, with mRNA expression, methylation status. **(c)** Specific HMR (S1) leading to different expression at isoform level, example of *Trp63* in keratinocytes. **(d)** *Fat1* with high CG methylation at the annotated promoter region with expression in both cells, but have HMRs and RNA-seq signals at approximately 15 Kb upstream.

The TS-HMRs within 1 kb of the transcription start site positively correlate with gene expression in both cell types with exceptions (Figure 3a, b). Promoters in the C1 class of HMRs with two identical boundaries have variable expression levels in the two cell types with many (5%) being more expressed in the fibroblasts [see Additional file 2: Figure S12a]. Promoters in the C2 class with one common boundary and a longer fibroblast HMR (C2S/L) show an increase in expression (6%) in fibroblasts as seen for the C1 class [see Additional file 2: Figure S12b]. Many promoters in the C2 class that is longer in keratinocytes show increased expression (2%) [see Additional file 2: Figure S12c]. The more complex overlapping HMRs (C3-5) show even larger variations in gene expression with less correlation between HMR length and gene expression, highlighting the complex relationship between HMRs and gene expression [see Additional file 2: Figure S12d-h].

The UCSC browser shots highlight different examples of differential methylation and gene expression (Figure 3c-d, [see Additional file 2: Figure S13-15]). Figure 3c shows the isoform level expression of epidermal specific gene P63 (*Trp63*) in keratinocyte is mediated by the TS-HMR at an alternative promoter [39]. Similar results are also observed for fibroblast-specific isoform level expression of *Arap1* [see Additional file 2: Figure S13a]. Gene expression for 64 genes with differentially methylated exons within CGIs [see Additional file 2: Figure S16] shows more highly expressed genes with methylated CGIs highlighted by *Hoxc13* [see Additional file 2: Figure S13c] [24], while a few are highly expressed when the CGIs are unmethylated. These unmethylated CGIs are at the first or second exons and are essentially extended HMRs like CGI shores highlighted by *Hya12* [see Additional file 2: Figure S13d] [22, 29]. Methylated exons with methylated conserved CGs are more highly expressed than conserved unmethylated exons [see Additional file 2: Figure S17-18].

*Fat1* shows high expression in both fibroblasts and keratinocytes with a methylated UCSC genome browser annotated promoter (Figure 3d). Closer evaluation of this gene shows that approximately 15 kbp upstream of the annotated TSS of *Fat1*, there is a HMR at a CGI, and RNA-seq data from paired-end 102-bp reads identify transcripts between the annotated promoter and the upstream HMR, suggesting a misannotation of this promoter; it is probable that the TSS of this gene is at the upstream HMR at the CGI, or that it is a new splicing isoform of *Fat1* (Figure 3d, [see Additional file 2: Figure S19]).

### Transcription factor binding sites enriched in common and tissue-specific hypomethylated regions

We evaluated the enrichment of transcription factor binding sites (TFBS) in the overlapping and TS-HMRs using two methods. We used support vector machines (SVM) [40, 41] to determine the enriched motifs in keratinocyte and fibroblast TS-HMRs [see Additional file 2: Figure S20, S21]. The second method is an enrichment calculation with respect to the whole genome. We searched for enriched DNA motifs among the 935 position weight matrices (PWMs) collected from the TRANSFAC databases [42] in five classes of HMRs: fibroblast-specific HMRs, keratinocyte-specific HMRs, HMRs common to keratinocytes and fibroblasts and the tissue-specific extensions of overlapping HMRs in fibroblast and keratinocytes. Distinct classes of motifs are enriched in each cell type. *P63*, *P53* and *TFAP2* are enriched in keratinocytes specific HMRs [see Additional file 2: Figure S20a] and are significantly expressed in this cell type (Figure 3c, [see Additional file 2: Figure S21a]). In dermal fibroblasts, *ELK1*, *E2F1*, *CREB*, *CREBP*, *ETS* motifs are prominently enriched [see Additional file 2: Figure S20a], and the mRNA for the transcription factors (*ELK1*, *E2F1*, *CREB3l1* and *CREB3l2*) that bind these motifs are significantly expressed [see Additional file 2: Figure S21a]. Examining the tissue-specific extensions of common HMRs (C2-C5), shows that the motifs enriched are similar to those in the tissue-specific HMRs [see Additional file 2: Figure S20b-c, S21b-g]. The common HMRs are enriched for *CNOT3*, *ZF5*, *E2F1*, *AP2* and *ETF* [see Additional file 2: Figure S20d, S21b-g].

### C/EBPβ binds in methylated regions

**Figure 4:**
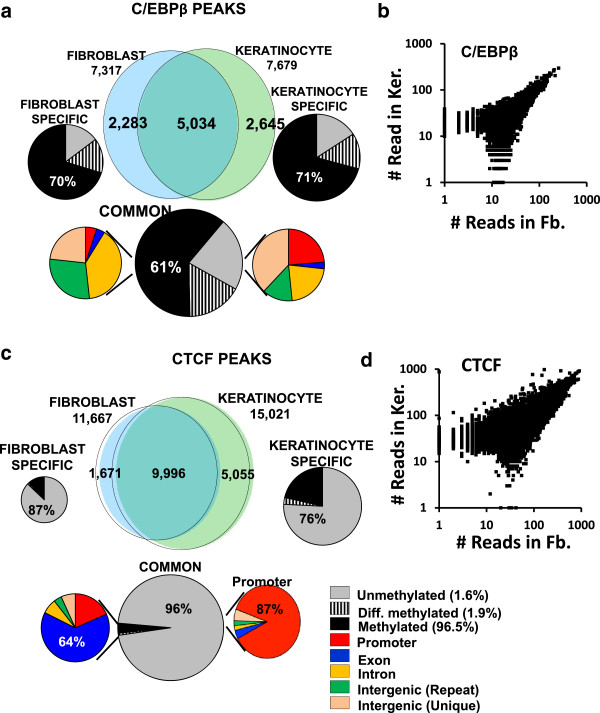
**C/EBPβ and CTCF chromatin immunoprecipitation (ChIP)-seq peaks in fibroblasts and keratinocytes. (a)** Venn diagram showing C/EBPβ ChIP-seq peaks in fibroblasts and keratinocytes. The common peaks are in methylated regions (61%) and tissue-specific C/EBPβ peaks are even more enriched in methylated regions (approximately 70%). **(b)** Scatter plot of number of reads in C/EBPβ bound ChIP peaks in dermal fibroblasts and keratinocytes. **(c)** Venn diagram showing that the majority of CTCF ChIP-seq peaks overlap between fibroblast and keratinocyte. 96% of the common peaks are in unmethylated regions and enriched in the promoters (87%). The tissue-specific CTCF peaks are also primarily in unmethylated regions enriched for promoters. The 3% of common CTCF peaks in methylated regions tend to occur in exons (64%). **(d)** Scatter plot of number of reads in CTCF bound ChIP peaks in dermal fibroblasts and keratinocytes.

**Figure 5:**
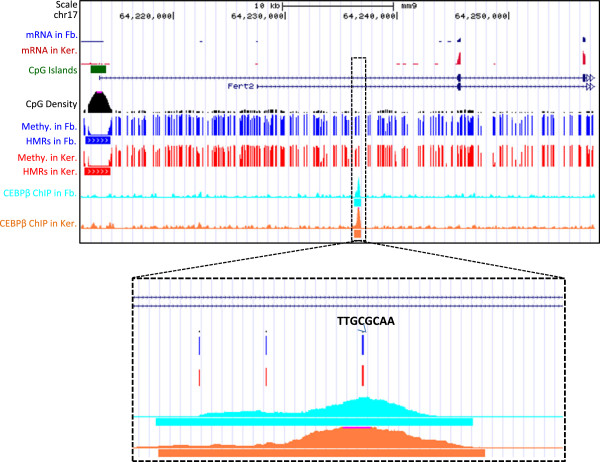
**C/EBPβ binding to the methylated canonical C/EBP motifs (TTGCGCAA).** UCSC genome browser screen shots as example for C/EBPβ binding to the methylated canonical C/EBP motif of TTGCGCAA in both fibroblasts and keratinocytes.

We examined the localization of two TFs using ChIP-seq, one that preferably binds methylated motifs (C/EBPβ) outside of HMRs and a second that preferably binds unmethylated motifs (CTCF) in HMRs. C/EBPβ is a B-ZIP transcription factor that preferentially binds methylated motifs [8, 31]. C/EBPβ ChIP-seq data identified 7,317 and 7,679 peaks in dermal fibroblasts and keratinocytes with approximately 2/3 overlapping (Figure 4a, b). The overlapping and tissue-specific C/EBPβ peaks are distributed in the genome similarly with approximately 2/3 in methylated regions, approximately 1/6 in unmethylated regions and approximately 1/6 in regions of differential methylation (Figure 4a). Among the commonly bound C/EBPβ peaks, 423 C/EBPβ bound sites are in unmethylated promoter regions (Figure 4a, [see Additional file 1: Table S6-7]). The motifs in the commonly bound peaks are mainly the consensus C/EBP sites that contain a central CG dinucleotide or its deaminated TG/CA dinucleotide, which is more abundant [see Additional file 2: Figure S22a]. Fibroblast-specific C/EBPβ ChIP-seq peaks shows enrichment for *AP1* and *SP1* motifs in addition to the consensus CEBP sites, while for keratinocytes, we only observe enrichment for the C/EBP motif [see Additional file 2: Figure S22a]. Examining tissue-specific C/EBPβ peaks that are differentially methylated does not identify a simple relationship between methylation and binding [see Additional file 1: Table S6-7], even though methylation enhances C/EBPβ binding *in vitro*[8, 31]. Figure 5 presents an example of C/EBPβ binding to a methylated canonical C/EBP motif (TTGC|GCAA). Several global C/EBP localization data sets have been published [43, 44], but none has highlighted C/EBPβ localization in the methylated regions of the genome.

### CTCF binds in hypomethylated regions

CTCF is a zinc finger protein initially known for its insulating properties at the beta globin locus [45–47]. More recent work has identified CTCF primarily at unmethylated regions of the DNA [19, 48]. CTCF ChIP-seq data identified approximately 12,000 peaks in fibroblasts and approximately 16,000 peaks in keratinocytes with approximately 10,000 being common (Figure 4c, d). Among the common CTCF peaks between these two cell types, approximately 96% of CTCF peaks are in HMRs, which are primarily promoters (87%) in CGIs (83%) [see Additional file 1: Table S6-7]. A total of 3% of the CTCF peaks that are in methylated regions and the less than 1% that are differentially methylated are enriched in exons (64%) [49]. The tissue-specific CTCF peaks that are in methylated regions (12% in fibroblasts and 21% in keratinocytes) also tend to occur in exons [see Additional file 1: Table S6-S7]. The motifs in the commonly bound peaks are mainly the consensus *CTCF*, *KLF, SP1* and *SP2* sites, as previously reported [46, 50], which are GC rich and contain CG dinucleotide [see Additional file 2: Figure S22b]. Fibroblast-specific CTCF ChIP-seq peaks show enrichment for *ZEB1* motif in addition to the consensus *CTCF* sites, while for keratinocytes, we observe that the enriched motifs are similar to the common bound peaks [see Additional file 2: Figure S22b]. An examination of the tissue-specific peaks that are differentially methylated identifies no simple relationship, even though it has been reported that CG methylation inhibits CTCF binding [19, 48].

## Discussion

We used bisulfite-based MethyC-seq technology to determine the genome-wide single nucleotide cytosine methylation maps for newborn female mouse primary dermal fibroblasts [31] and keratinocytes and identified hypomethylated regions (HMRs) [18] in both cell types. Out of 1.4 million completely unmethylated CGs in the primary keratinocytes, 72% are in HMRs and represent 2.9% of the genome, indicating the clustered nature of unmethylated CG dinucleotides in the mouse genome. This is reflected in the correlation of CG methylation among the first and second neighboring CGs [see Additional file 2: Figure S2]. Methylated cytosines are prone to deamination and often are less conserved in the genome [6, 51, 52]; however, methylated CGs in exons showed more conservation than the unmethylated CGs in exons highlighting their potential importance [24]. Comparing HMRs from the two cell types identified overlapping HMRs and tissue-specific HMRs. Overlapping HMRs tend to be longer and enriched in promoters containing CGIs, compared to the TS-HMRs, which tend to be shorter and not in promoters. Overlapping HMRs were also unmethylated in two additional (ES and NP) mouse methylomes, suggesting housekeeping functions. Both the overlapping and TS-HMRs are more CG rich and evolutionarily conserved than the surrounding regions. Comparing the two methylomes allowed the identification of four classes of CG dinucleotides. These methylation frequencies are a trait of a population of cells, not an individual cell, and the mechanisms that can produce CG dinucleotides with four distinct methylation profiles highlight the dynamic nature of CG methylation.

Different TFBSs are enriched in the common HMRs, the keratinocyte TS-HMRs, and the fibroblast TS-HMRs. The TFs that bind the TFBSs enriched in the common HMRs are expressed in both cell types, while the TFs that bind TFBS enriched in the fibroblast TS-HMRs are only expressed in the fibroblast, as is also true for keratinocytes. The TFBSs that are enriched in the unique TS-HMRs are also enriched in the tissue-specific portion of the overlapping HMRs. Among the top keratinocyte-specific TFBSs, *P53* and *P63* binding sites do not necessarily contain a CG in their consensus binding site and should not be regulated by methylation. However, methylation of the *AP2α* motif is reported to inhibit DNA protein binding [53, 54]. The top five transcription factors enriched in common HMRs contain CG in their binding sites and methylation is known to inhibit DNA binding of several TFs [55]. Methylation inhibits binding to *E2F* TFBS for all family members of *E2F*[56]. Methylated *AP2* and *Zf5* sites have also been reported to inhibit DNA protein binding by *AP2* and *Zf5* TFs [53, 57]. The mechanism that connects TFs with HMRs is an important question to examine going forward.

Comparing the HMRs with mRNA-seq data for both cell types identified several general patterns. We identified examples of TS-HMRs at promoters where unmethylation positively correlates with gene expression. Classic examples in keratinocytes are *P63*, *keratin1*, and *keratin5* and in fibroblasts are *ARAP1*, and *NNMT* (Figure 3c, [see Additional file 2: Figure S13a, S14]). We also identified examples of HMRs being extended toward the body of the gene and demethylation towards the gene body positively correlating with gene expression. This is analogous to what was observed for CGI shores [22]. Another pattern is methylation at the last exon that positively correlates with gene expression [see Additional file 2: Figure S13c-d] [15, 24, 33]. HMRs are not always associated with gene expression. For example, the longest HMR is identical in both cell types and overlaps with the *Hoxb13* gene [see Additional file 2: Figure S5c]. This gene is not expressed in either cell type, suggesting some other mechanism, for example activating histone marks is required for the activation of this gene.

ChIP-seq for C/EBPβ and CTCF highlight the two parts of the genome, the HMRs representing 2 to 3% of the genome and the remaining methylated genome. A total of 96% of CTCF peaks are in HMRs, while 70% of the C/EBPβ peaks are in the methylated regions. CTCF is present in several promoters with HMRs, while the C/EBPβ binding to the methylated sites is mostly enriched in introns and intergenic regions. Evaluating the genome-wide binding of these two transcription factors clearly shows that there are two parts to the genome and mechanisms of how these two parts communicate needs to be explored in more detail.

## Conclusions

Keratinocytes and fibroblasts are of epithelial and mesenchymal origins, two of the three primary germ layers. Primary dermal fibroblasts cultures in the presence of serum showed a gene-expression pattern very similar to that of oncogene expressing cancer cells. In contrast, epidermal keratinocytes express tumor suppressor genes. However, in dermal fibroblasts, we showed a different mechanism of gene regulation at these genes. CGIs at these two cell types are largely invariable; however, demethylation at the CG-shores or HMR-shores toward the gene body is shown to be correlated with the expression of these genes. Additionally, many non-CGI HMRs at the alternative promoters are shown to regulate the expression of tumor suppressor or oncogenes suggesting an epigenetic regulation of gene expression beyond CGIs. TS-HMRs have less methylation than surrounding DNA in all four methylomes examined, suggesting a dynamic methylation-demethylation process at these regulatory regions [58]. These high-resolution methylation maps and the RNA-seq data in these two primary cell types can be used as reference methylomes and transcriptomes for evaluating both pathological methylomes and differentiation of stem cell [59].

## Methods

### Mouse primary keratinocytes and dermal fibroblasts

NIH research guidelines and IACUC-approved animal study protocols were followed in this study. Keratinocytes and dermal fibroblasts were cultured from wild-type newborn mice according to the protocol described previously (Rishi *et al*. [8]). Primary keratinocytes were seeded at a density of one mouse epidermis per 10-cm dish or equivalent in calcium- and magnesium-free SMEM (GIBCO Laboratories, Grand Island, NY, USA), supplemented with 8% Chelex (Bio-Rad, Richmond, CA, USA)-treated FBS (Atlanta Biologicals, Inc) and 0.2 mM calcium (CaCl2). Dermal fibroblasts were also seeded at a density of one mouse dermis per 10-cm dish or equivalent in DMEM/F12: GlutaMAX medium (Invitrogen, USA) with 10% FBS.

### RNA-sequencing in keratinocytes

Total RNA was isolated from the mouse primary keratinocytes and dermal fibroblasts. Purified RNA was used for generating the mRNA-seq library using the Illumina mRNA-seq kit as described in the manufacturer's protocol. Data analysis was performed using Partek Genomic suite with the default parameters. Transcript abundances were reported in RPKM (reads per kilobase per million mapped reads) with arbitrary units.

### Determination of whole genome DNA methylation in keratinocytes

Genomic DNA was isolated from primary keratinocytes that had been cultured for 3 days, and bisulfite sequencing was used according to the protocol described previously [31]. Approximately 10 μg of genomic DNA was sonicated to approximately 300 bp using the Covaris S2 System. Sonicated DNA was purified using Qiagen DNeasy MinElute columns (Qiagen Inc., USA). Each sequencing library was constructed using the Illumina paired-end DNA sample preparation kit (Illumina Inc., USA) according to the manufacturer's instructions, with the following modifications: Illumina methylated adapters were used in place of the standard genomic DNA adapters. Ligation products were purified with AMPure XP beads (Beckman Coulter, USA). 4 × 500 ng of DNA were bisulfite-treated using the EpiTect Bisulfite Kit (Qiagen Inc., USA) following the manufacturer's guidelines, followed by PCR amplification using the Phusion Taq using the following PCR conditions: 2 min at 95°C, 4 cycles of 15 sec at 98°C, 30 sec at 60°C, 4 min at 72°C, and 10 min at 72°C. Libraries were sequenced using the Illumina HiSeq 2000 (Illumina Inc., USA) up to 101 cycles. Mapping the bisulfite-treated reads was done with methods described previously [31] with tools from Novoalign and Novomethyl (Novocraft Technologies, http://www.novocraft.com/) packages. Hypomethylated regions (HMRs) were identified with a hidden Markov model (HMM) as described previously [18]. The false positive rate of the maps of bisulfite sequencing reads was calculated two ways: one, considering the non-CG cytosine methylation and second, considering the chromosome Y cytosine methylation because we used female mouse to determine these two methylomes. Although, non-CG methylation is observed in the ES cells and chromosome Y has some homologies to the chromosome X, two things that might cause a false estimation of the calculation, the false positive rate of these two methylomes is considerably low.

### Chromatin immunoprecipitation sequencing of C/EBPβ and CTCF

C/EBPβ and CTCF chromatin immunoprecipitation (ChIP) sequencing from primary dermal fibroblasts and keratinocytes were done as described previously [31]. Briefly, primary cultured dermal fibroblasts and keratinocytes were chemically cross-linked for 10 min by adding 0.6% formaldehyde (Sigma, USA) directly to the medium. The cross-linking reaction was stopped by adding 125 mM glycine, and dishes were swirled for 5 min at room temperature. Cells were washed twice with ice-cold PBS and harvested in ice-cold PBS containing protease inhibitor (Roche, USA). A total of 107 cells were pelleted by centrifugation at 4°C for 5 min at 300 g. Four times, 300 μl of sonicated chromatin preparation was incubated overnight with C/EBPβ (sc-150; Santa Cruz) or CTCF antibody (sc-15914; Santa Cruz). Immunocomplexes were captured using protein G agarose beads (Invitrogen Inc., USA) and washed twice with the buffer containing 2 mM EDTA, 100 mM Tris-Cl, pH 8.0, and 0.18% Sarkosyl, and four times with the IP buffer (100 mM Tris-Cl, pH 8.5, 500 mM LiCl, 1% NP40, 1% deoxycholic acid). After being incubated with RNaseA and Proteinase K, DNA was eluted using a QIAquick PCR Purification Kit (Qiagen, Germany). Purified DNA were used to prepare the library for Illumina high-throughput sequencing using Illumina Single End ChIP-seq Sample Preparation Kit, as described in the manufacturer's protocol. Libraries were sequenced to generate 35-bp single-end reads using Illumina GAII sequencing machines. We used the Model-Based Analysis of ChIP-seq (MACS) algorithm [60] with default parameters for detecting the ChIP-seq peaks of C/EBPβ and CTCF.

### Calculation of motif enrichment in tissue-specific and common hypomethylated regions

To determine the enriched motifs in tissue-specific and common HMRs, we calculated an enrichment score for each motif. To avoid the bias of sampling from the mouse genome, we searched each motif on the whole genome. In each chromosome, motifs were searched using MAST in MEME suite [61] with the position weight matrices (PWMs). The PWMs we used were collected from the TRANSFAC databases [42] in which 935 PWMS are provided, and MAST was run with default parameters. For each motif *M* with the length *L* we denote *M*(*x*_*start*_*:x*_*end*_) to record the positions where the motif starts and ends: *x*_*1*_*:x*_*1*_ *+ L-1, x*_*2*_*: x*_*2*_ *+ L-1 … x*_*N*_*: x*_*N*_ *+ L-1*, *N* being the total number of motifs in genome. For each position *x*_*i*_*: x*_*i*_ *+ L-1*, if it overlapped with the examined regions (HMRs), *x*_*i*_ = 1, otherwise *x*_*i*_ = 0. For whole HMRs, the observed (*OCC*_*obs*_) and expected (*OCC*_*exp*_) occurrences of the motif are calculated as:  and , where *N* is the total number of motif in the whole genome, *L*_*r*_ is the total length of base pairs in the examined regions (HMRs), and *L*_*g*_ is the total length of base pairs for the whole mouse genome. The enrichment score (*E*) for motif *M* is calculated as following: , where *OCC*_*obs*_ is the observed occurrences, and *OCC*_*exp*_ is the expected occurrence of motif *M* in examined regions (tissue-specific or common HMRs).

UCSC annotations of exon, intron, 5'UTRs, 3'UTRs, and promoters (0 to -2 kbps) were used for the analysis. UCSC genome browser screen shots were generated using custom tracks of the UCSC web site (https://genome.ucsc.edu/).

### Data submission

Keratinocyte methylome data have been submitted to the GEO database with accession number (GSE44918). Two biological replicates of keratinocytes mRNA-seq data, C/EBPβ and CTCF ChIP-seq data will be submitted to the GEO database (GSE44918). Fibroblasts methylome, C/EBPβ ChIP-seq and mRNA-seq data have been obtained from the GEO accession number (GSE44942) [31]. The CTCF ChIP-seq data and the second biological replicate RNA-seq data of dermal fibroblasts will be submitted to the GEO database (GSE44942). The data can also be obtained from the authors upon request.

## Electronic supplementary material

Additional file 1: Table S1: Sequencing reads and aligned reads. **Table S2.** Statistics of methylated cytosines. **Table S3.** Repeats in UCSC repeat masker. **Table S4.** HMRs overlap with CpG Islands. **Table S5.** Fb and Ker mRNA GO analysis. **Table S6.** Total number of unmethylated and methylated tissue specific and common peaks of C/EBPβ and CTCF. **Table S7.** Distribution of unmethylated and methylated tissue specific and common peaks of C/EBPβ and CTCF based on CGI and non-CGI. (PDF 438 KB)

Additional file 2: Figure S1: Methylation of CGs and coverage in fibroblasts and keratinocytes. **Figure S2.** Adjacent CGs have a similar methylation status. **Figure S3.** Comparison of CG methylation in different repetitive elements in fibroblasts and keratinocytes. **Figure S4.** Methylated CGs at exons are more conserved than the unmethylated CGs. **Figure S5.** HMRs in fibroblasts and keratinocytes. **Figure S6.** Overlap with CGIs and composition of different methylated CGs in tissues-specific HMRs (S1) and common HMRs (C1-C5). **Figure S7.** Heatmap of CG methylation for the different groups of keratinocyte HMRs in different cells. **Figure S8.** Comparison of HMRs in fibroblasts, keratinocytes, embryonic stem cells and neuronal progenitor cells. **Figure S9.** HMRs are the conserved regions with high GC content as well as CpG density. **Figure S10.** SINE elements are depleted inside the HMRs, but enriched in the surrounding regions; however, both LTR and LINE elements are depleted in both inside and at the surrounding regions. **Figure S11-S12.** Comparison of mRNA expression between fibroblasts and keratinocytes. **Figure S13.** UCSC genome browser screen shots as examples for different types of HMRs. **Figure S14.** UCSC genome browser screen shots as examples for tissue specific genes expression with TS-HMRs near the TSS of *Krt1*, *Krt5* and *NNMT1*. **Figure S15.** UCSC genome browser screen shots as examples for extended HMRs leading to enhanced gene expression of *Emilin1*, *Trp53il1* and *Wnt3*. **Figure S16.** Correlation between the methylation difference and gene expression changes in fibroblasts and keratinocytes. **Figure S17.** Exons with methylated conserved CGs are highly expressed. **Figure S18.** Exons with methylated conserved CGs are highly expressed. **Figure S19.** RNA-seq signal at the *Fat1* locus. **Figure S20.** Transcription factor binding sites (TFBS) in HMRs. **Figure S21.** Comparison of enriched TFBS motifs in the fibroblasts and keratinocyte HMRs. **Figure S22.** Binding motifs in C/EBPβ and CTCF ChIP-seq peaks. (PDF 4 MB)

## Abbreviations

CDS: Coding sequence
CGI: CG Island
ChIP: Chromatin immunoprecipitation
EMT: Epithelial to mesenchymal transition
ES: Embryonic stem cell
HMM: Hidden Markov model
HMR: Hypomethylated region
LINE: Long interspersed element
LTR: Long terminal repeat
NP: Neuronal progenitor cells
PWM: Position weight matrices
RPKM: Reads per kilobase per million mapped reads
SINE: Short interspersed element
SVM: Support vector machines
TFBS: Transcription factor binding sites
TS-HMR: Tissue-specific hypomethylated region
TSS: Transcription start site
UTR: Untranslated region.
