# Supplementary material for: High-resolution genome-wide DNA methylation maps of mouse primary female dermal fibroblasts and keratinocytes
Source: Epigenetics Chromatin. 2014 Dec 2;7:35. doi: 10.1186/1756-8935-7-35 (PMC4333159; doi:10.1186/1756-8935-7-35)
Supplement: Supplementary file 1 — Additional file 1: Table S1: Sequencing reads and aligned reads. Table S2. Statistics of methylated cytosines. Table S3. Repeats in UCSC repeat masker. Table S4. HMRs overlap with CpG Islands. Table S5. Fb and Ker mRNA GO analysis. Table S6. Total number of unmethylated and methylated tissue specific and common peaks of C/EBPβ and CTCF. Table S7. Distribution of unmethylated and methylated tissue specific and common peaks of C/EBPβ and CTCF based on CGI and non-CGI. (PDF 438 KB) [file 13072_2014_350_MOESM1_ESM.pdf]

Table S1: Sequencing Reads and aligned reads

a. Primary Dermal fibroblasts

Lane	Total Reads	Aligned reads	% Alignment
1_s_2	267,656,800	180,171,584	67.31
1_s_3	265,985,560	178,042,371	66.94
1_s_4	237,590,528	165,256,556	69.56
1_s_5	267,421,542	178,495,712	66.75
1_s_6	272,517,984	180,762,840	66.33
1_s_7	269,590,532	176,227,208	65.37
1_s_8	276,364,664	182,947,476	66.20
0_s_1	233,865,622	156,924,697	67.10
0_s_2	235,188,988	159,500,249	67.82
0_s_3	238,428,236	165,914,005	69.59
0_s_4	237,590,528	165,256,556	69.56
0_s_5	235,318,182	162,436,258	69.03
0_s_6	236,538,180	159,061,975	67.25
0_s_7	237,196,222	164,957,203	69.54
0_s_8	236,003,884	164,823,395	69.84
**Total**	**3,747,257,452**	**2,540,778,085**	**67.80**

b. Primary Keratinocytes

Lane	Total Reads	Aligned reads	% Alignment
1_s_2	196,589,570	134,532,460	68.43
1_s_3	197,130,122	134,680,829	68.32
1_s_4	195,554,382	132,422,537	67.72
1_s_5	186,719,100	127,937,421	68.52
1_s_6	196,221,464	133,404,387	67.99
1_s_7	184,363,808	125,768,513	68.22
1_s_8	183,304,880	124,754,236	68.06
0_s_3	38,933,004	25,454,112	65.38
**Total**	**1,378,816,330**	**938,954,495**	**68.10**

c. CpG average coverage and methylation

	Total # genomic CpG	Average coverage	Average methylation	FDR (%)
**Fibroblasts**	21,342,779	91	65	0.007
**Keratinocytes**	21,342,779	36	67	0.004
**Fibroblasts 5 lanes**	21,342,779	32	65	0.004

d. The CpG methylation in unique and repeat regions.

	#	%
**Total CpGs**	21,342,779	100.0%
**Covered by Fb. Methylome**	19,906,237	93.3%
**Covered by Ker. Methylome**	19,789,633	92.7%
**Unique Regions**	13,132,502	61.5%
**Covered by Fb. Methylome**	12,914,077	98.3%
**Covered by Ker. Methylome**	12,896,130	98.2%
**Repetative Regions**	8,210,277	38.5%
**Covered by Fb. Methylome**	6,992,160	85.2%
**Covered by Ker. Methylome**	6,893,503	84.0%

Table S2: Statistics of methylated Cytosines

a. Statistics of Methylated Cs in Ker 36X

	CT Strand			GA Strand		
	Total	Unmethylated	Methylated	Total	Unmethylated	Methylated
CpG	19292222	4,398,099	14894123	19321822	4,403,335	14918487
CHG	104671688	104,609,887	61801	104644114	104,579,881	64233
CHH	351161524	350,969,181	192343	351195535	350,999,886	195649
total	475125434	459977167	15148267	475161471	459983102	15178369

b. Totally unmethylated CGs in Fb. and Ker.

	M=0 Total	#M=0 in other Cell	#M=1 in Other Cell
**Fb.**	1,591,241	1,436,343	16,195
**Ker.**	1,965,497	1,436,343	8,916

c. Totally unmethylated CGs in HMRs

	M=0 Total	#M=0 in HMRs	%M=0 in HMRs
**Fb.**	1,591,241	1,037,641	65.2%
**Ker.**	1,965,497	1,409,942	71.7%

d. Statistics of methylation status of each CG in the HMRs

Methylation	Total	# in S1	% in S1	# in HMR	% in HMR
			**Fb.**		
**<10%**	2,141,857	35,022	1.6%	1,539,769	71.9%
**10%~50%**	2,340,863	78,438	3.4%	449,157	19.2%
**>50%**	15,423,517	8,135	0.1%	23,233	0.2%
			**Ker.**		
**<10%**	2,452,276	158,770	6.5%	1,803,994	73.6%
**10%~50%**	1,813,608	219,714	12.1%	521,112	28.7%
**>50%**	15,523,749	21,453	0.1%	46,330	0.3%

e. Statistics of methylation status of each CG in the HMRs

Types	Fb.			Ker.		
	<10%	10%~50%	>50%	<10%	10%~50%	>50%
**S1**	35,022 (28.8%)	78,438 (64.5%)	8,135 (6.7%)	158,770 (39.7%)	219,714 (54.9%)	21,453 (5.4%)
**C1**	262,601 (84.6%)	45,459 (14.6%)	2,309 (0.7%)	271,971 (87.7%)	35,110 (11.3%)	2,982 (1.0%)
**C2S**	469,997 (83.7%)	88,530 (15.8%)	3,016 (0.5%)	193,062 (84.0%)	33,987 (14.8%)	2,744 (1.2%)
**C2L**	192,276 (78.1%)	50,945 (20.7%)	2,861 (1.2%)	513,093 (85.5%)	80,447 (13.4%)	6,746 (1.1%)
**C3**	149,951 (75.5%)	46,496 (23.4%)	2,243 (1.1%)	160,488 (79.9%)	37,140 (18.5%)	3,284 (1.6%)
**C4S**	270,848 (80.2%)	65,014 (19.2%)	1,973 (0.6%)	41,688 (77.3%)	11,290 (20.9%)	946 (1.8%)
**C4L**	44,072 (68.8%)	19,140 (29.9%)	831 (1.3%)	324,562 (82.5%)	63,577 (16.2%)	5,273 (1.3%)
**C5S**	80,963 (67.8%)	37,248 (31.2%)	1,287 (1.1%)	31,521 (69.1%)	13,137 (28.8%)	973 (2.1%)
**C5L**	34,039 (64.8%)	17,887 (34.1%)	578 (1.1%)	108,839 (79.2%)	26,710 (19.4%)	1,929 (1.4%)
**Total**	1,539,769 (76.5%)	449,157 (22.3%)	23,233 (1.2%)	1,803,994 (76.1%)	521,112 (22.0%)	46,330 (2.0%)

Table S3: Repeats in UCSC repeat masker

Repeat name	# Repeats	Max Length	Min Length	Average Length	Median Length	Upstream of Ker TS-HMR (same size) 27,370 (Random: 27,656)	Inside 15,665 (Random: 28,092)	Down stream (same size) 27,539 (Random: 28,118)
SINE	1,505,855	517	11	134.64	136	11,584 (8,029)	5,781 (8,135)	11,709 (8,094)
Simple repeat	1,037,272	3,444	6	61.22	45	6,257 (5,124)	3,755 (5,201)	6,386 (5,286)
LINE	942,656	13,492	11	553.23	280	2,723 (6,434)	1,739 (6,682)	2,720 (6,621)
LTR	824,426	7,752	11	334.68	279	3,627 (5,191)	2,235 (5,174)	3,662 (5,181)
Low complexity	369,568	1,645	11	53.16	37	1,754 (1,749)	1,122 (1,781)	1,687 (1,831)
DNA repeats	153,284	2,178	11	182.48	162	1,146 (846)	878 (831)	1,141 (847)
Other	17,033	2,134	11	427.37	313	67 (123)	26 (108)	39 (115)
scRNA	8,152	166	11	78.95	81	72	40	58
Unknown	7,667	2,945	11	204.04	132	48	26	34
Satellite	4,825	38,417	11	258.30	141	35	21	35
tRNA	4,749	108	11	61.68	64	28	16	30
snRNA	3,003	214	11	81.98	84	16	13	19
rRNA	1,559	1,849	16	108.46	86	8	12	11
RNA	691	339	12	171.41	339	4	3	2
Remaining other	418	741	16	184.32	160	-	-	-
srpRNA	284	359	12	130.21	115	2	0	3

Table S4: HMRs overlap with CpG Islands

HMRs	Total	UCSC.CGIs (15,991)		Bird.CGIs (23,021)		CpGProD (41,359)	
		#	%	#	%	#	%
**Ker HMRs**	71,495	13,460	18.8%	17,480	24.4%	17,239	24.1%
**Fb HMRs**	49,233	13,520	27.5%	17,390	35.3%	16,979	34.5%
**Ker Specific HMRs**	34,967	143	0.4%	545	1.6%	910	2.6%
**Fb Specific HMRs**	12,091	46	0.4%	182	1.5%	367	3.0%
**Ker and Fb Common HMRs**	37,142	13,474	36.3%	17,208	46.3%	16,612	44.7%
**Ker+Fb+ESC+NP Common HMRs**	21,054	12,839	61.0%	15,548	73.8%	14,974	71.1%

Table S5: Fb and Ker mRNA GO analysis

a. GO enrichment for genes specifically expressed in dermal fibroblasts

Term	Name	Count	P-Value	Fold Enrichment	Bonferroni
GO:0007155	cell adhesion	49	4.9E-18	4.36	7.2E-15
GO:0030198	extracellular matrix organization	21	7.7E-15	10.39	1.1E-11
GO:0016064	immunoglobulin mediated immune response	12	4.0E-08	9.52	5.9E-05
GO:0009611	response to wounding	24	4.8E-07	3.46	7.0E-04
GO:0002250	adaptive immune response	12	8.3E-07	7.14	1.2E-03
GO:0006955	immune response	28	8.3E-07	2.97	1.2E-03
GO:0002252	immune effector process	13	8.0E-06	5.15	1.2E-02
GO:0006952	defense response	24	3.3E-05	2.68	4.8E-02
GO:0006954	inflammatory response	16	4.6E-05	3.55	6.6E-02
GO:0006935	chemotaxis	12	1.1E-05	5.50	1.6E-02
GO:0010811	positive regulation of cell-substrate adhesion	7	1.2E-05	12.95	1.8E-02
GO:0001501	skeletal system development	24	1.3E-08	4.21	1.9E-05
GO:0009792	embryonic development ending in birth or egg hatching	23	4.3E-05	2.70	6.2E-02
GO:0048705	skeletal system morphogenesis	12	5.8E-05	4.61	8.2E-02
GO:0001568	blood vessel development	17	3.1E-05	3.48	4.5E-02

b. GO enrichment for genes specifically expressed in Keratinocytes

Term		Count	P-Value	Fold Enrichment	Bonferroni
GO:0031424	keratinization	13	1.4E-16	38.70	1.1E-13
GO:0008544	epidermis development	20	4.2E-16	13.34	4.3E-13
GO:0007398	ectoderm development	20	1.5E-15	12.54	1.4E-12
GO:0030216	keratinocyte differentiation	14	8.1E-15	24.31	7.8E-12
GO:0009913	epidermal cell differentiation	14	1.9E-14	22.88	1.9E-11
GO:0060429	epithelium development	23	1.1E-12	7.08	1.1E-09
GO:0030855	epithelial cell differentiation	17	1.2E-12	11.52	1.2E-09
GO:0007155	cell adhesion	26	1.2E-08	3.86	1.1E-05
GO:0032355	response to estradiol stimulus	4	3.2E-05	55.57	3.1E-02
GO:0048729	tissue morphogenesis	14	5.4E-06	4.90	5.2E-03

Table S6: Total number of unmethylated and methylated tissue specific and common peaks of C/EBPβ and CTCF

	CTCF binding				C/EBPb binding			
	Common (n=9,996)				Common (n=5,034)			
**Fibroblast**	**U**	**U**	**M**	**M**	**U**	**U**	**M**	**M**
**Keratinocytes**	**U**	**M**	**U**	**M**	**U**	**M**	**U**	**M**
	96%	0%	1%	3%	22%	10%	6%	61%
**Total no. of peaks (n)**	(9.627)	(2)	(50)	(307)	(1,108)	(506)	(327)	(3,093)
**Promoter**	87%	17%	20%	18%	24%	8%	6%	4%
**Exon**	4%	42%	66%	64%	3%	4%	3%	4%
**Intron**	2%	8%	8%	7%	22%	36%	39%	40%
**Intergenic_Repeat**	2%	0%	0%	5%	14%	25%	24%	29%
**Intergenic_Unique**	5%	33%	6%	6%	38%	26%	28%	22%
	Fibroblast specific (n=1,671)				Fibroblast specific (n=2,283)			
**Fibroblast**	**U**	**U**	**M**	**M**	**U**	**U**	**M**	**M**
**Keratinocytes**	**U**	**M**	**U**	**M**	**U**	**M**	**U**	**M**
	87%	0%	1%	12%	15%	7%	8%	70%
**Total no. of peaks (n)**	(1,,455)	(1)	(16)	(199)	(336)	(168)	(174)	(1,605)
**Promoter**	84%	0%	19%	15%	51%	8%	11%	4%
**Exon**	3%	0%	50%	35%	1%	2%	6%	4%
**Intron**	4%	0%	0%	27%	19%	20%	40%	40%
**Intergenic_Repeat**	4%	0%	13%	16%	11%	20%	13%	27%
**Intergenic_Unique**	6%	100%	19%	8%	17%	33%	30%	25%
	Keratinocyte specific (n=5,055)				Keratinocyte specific (n=2,645)			
**Fibroblast**	**U**	**U**	**M**	**M**	**U**	**U**	**M**	**M**
**Keratinocytes**	**U**	**M**	**U**	**M**	**U**	**M**	**U**	**M**
	76%	1%	2%	21%	16%	7%	6%	71%
**Total no. of peaks (n)**	(3,830)	(36)	(107)	(1,082)	(420)	(194)	(157)	(1,874)
**Promoter**	77%	14%	27%	13%	51%	11%	10%	5%
**Exon**	6%	39%	43%	63%	3%	2%	3%	4%
**Intron**	5%	17%	13%	11%	16%	36%	47%	37%
**Intergenic_Repeat**	1%	3%	2%	5%	12%	20%	18%	31%
**Intergenic_Unique**	11%	28%	15%	8%	18%	30%	22%	23%

Table S7: **Distribution of unmethylated and methylated tissue specific and common peaks of C/EBPβ and CTCF based on CGI and non-CGI**

	CTCF binding								CEBPb binding							
	Common								Common							
	UU		UM		MU		MM		UU		UM		MU		MM	
	CGI	Non CGI	CGI	Non CGI	CGI	Non CGI	CGI	Non CGI	CGI	Non CGI	CGI	Non CGI	CGI	Non CGI	CGI	Non CGI
**Total**	9,135	492	12	0	31	19	214	93	271	837	0	506	0	327	1	3,092
**Promoter**	87%	86%	17%	-	19%	21%	18%	19%	1%	31%	-	8%	-	6%	0%	4%
**Exon**	4%	6%	42%	-	65%	68%	64%	66%	3%	3%	-	4%	-	3%	0%	4%
**Intron**	2%	2%	8%	-	10%	5%	6%	9%	6%	27%	-	36%	-	39%	100%	40%
**IG_repeat**	2%	1%	0%	-	0%	0%	5%	4%	0%	18%	-	25%	-	24%	0%	29%
**IG_unique**	5%	4%	33%	-	6%	5%	7%	2%	90%	21%	-	26%	-	28%	0%	22%

	Fibroblast-specific								Fibroblast-specific							
	UU		UM		MU		MM		UU		UM		MU		MM	
	CGI	Non CGI	CGI	Non CGI	CGI	Non CGI	CGI	Non CGI	CGI	Non CGI	CGI	Non CGI	CGI	Non CGI	CGI	Non CGI
**Total**	1,336	119	0	1	13	3	149	50	69	267	0	168	0	174	0	1,605
**Promoter**	86%	61%	-	0%	23%	0%	14%	16%	94%	40%	-	8%	-	11%	-	4%
**Exon**	3%	4%	-	0%	46%	67%	34%	38%	0%	2%	-	2%	-	6%	-	4%
**Intron**	3%	9%	-	0%	0%	0%	27%	26%	0%	24%	-	20%	-	40%	-	40%
**IG_repeat**	3%	12%	-	0%	15%	0%	17%	12%	3%	13%	-	20%	-	13%	-	27%
**IG_unique**	5%	14%	-	100%	15%	33%	8%	8%	3%	20%	-	33%	-	30%	-	25%

	Keratinocyte-specific								Keratinocyte-specific							
	UU		UM		MU		MM		UU		UM		MU		MM	
	CGI	Non CGI	CGI	Non CGI	CGI	Non CGI	CGI	Non CGI	CGI	Non CGI	CGI	Non CGI	CGI	Non CGI	CGI	Non CGI
**Total**	3,019	811	9	27	37	70	232	850	112	308	0	194	0	157	0	1,874
**Promoter**	84%	49%	0%	19%	30%	26%	18%	12%	95%	35%	-	11%	-	10%	-	5%
**Exon**	5%	10%	89%	22%	59%	34%	72%	61%	0%	4%	-	2%	-	3%	-	4%
**Intron**	3%	12%	0%	22%	3%	19%	3%	13%	1%	21%	-	36%	-	47%	-	37%
**IG_repeat**	1%	3%	0%	4%	0%	3%	0%	6%	2%	16%	-	20%	-	18%	-	31%
**IG_unique**	7%	26%	11%	33%	8%	19%	7%	8%	3%	23%	-	30%	-	22%	-	23%
