# Supplementary material for: High-resolution genome-wide DNA methylation maps of mouse primary female dermal fibroblasts and keratinocytes
Source: Epigenetics Chromatin. 2014 Dec 2;7:35. doi: 10.1186/1756-8935-7-35 (PMC4333159; doi:10.1186/1756-8935-7-35)

Additional file 2:

Figure Legends:

Figure S1. Methylation of CGs and coverage in fibroblasts and keratinocytes. (**a-b**) The histogram of percentage of CGs with methylation status at different coverage in (**a**) fibroblasts at 91X coverage and (**b**) keratinocytes. (**c-d**) The histogram of percentage of CGs with methylation status for each chromosome in (**c**) fibroblasts at 91X coverage and (**d**) keratinocytes. (**e**) The histogram of percentage of CGs with methylation status at different coverage in fibroblasts at 31X coverage. (**f**) Methylation status of all CGs in fibroblasts at 31X coverage. (**g-h**) Pie charts of composition of three types methylated Cs in (**g**) fibroblasts at 91X coverage and (**h**) in keratinocytes.

Figure S2: Adjacent CGs have a similar methylation status. (**a-b**) Pearson's correlation for methylation status of the first neighboring (**a**) and second neighboring (**b**) CpGs in fibroblasts. (**c-d**) Pearson's correlation for methylation status of the first neighboring (**c**) and secondneighboring (**d**) CpGs in keratinocytes.

Figure S3: Comparison of CG methylation in different repetitive elements in fibroblasts and keratinocytes. Average methylation of CGs in different repetitive elements: (**a**) LINE, (**b**) SINE, (**c**) LTR, (**d**) Simple Repeat, (**e**) Low complexity, (**f**) DNA repeats, (**g**) rRNA,(**h**) scRNA, (**i**) tRNA, (**j**) snRNA, (**k**) srpRNA, and (**l**) RNA. Fibroblasts are presented in blue and keratinocytes are in red.

Figure S4: Methylated CGs at exons are more conserved than the unmethylated CGs. (**a-f**) Distribution of unmethylated (0 to 10%) and methylated (90% to 100%) CpGs with varying PhyloP scores in Exons (**a-b**), Promoters (**c-d**) and Introns (**e-f**).

Figure S5: HMRs in fibroblasts and keratinocytes. (**a-b**) Length against number of CGs for HMRs in (**a**) fibroblasts and (**b**) keratinocytes. The length varies from 12 bps to 38,568 bps. The color for each HMR is coded according to the overlapping with fibroblast HMRs as shown in **Fig. 2C**. (**c-e**) UCSC genome browser screen shots for: (**c**) largest common HMR covered *Hoxb13* locus without expression in both fibroblasts and keratinocytes, (**d**) largest keratinocyte-

specific HMR covered *Mir205* locus with expression, and (**e**) largest fibroblast-specific HMR located in *Skint6* locus without expression.

Figure S6: Overlap with CGIs and composition of different methylated CGs in tissues-specific HMRs (S1) and common HMRs (C1-C5). The first and last three rows of the histogram show the percent of overlap with Bird. CGIs (experimental extracted CGIs from Dr. Bird group [1]), CpGProD (CGI predictions using CpGProD [2]), and UCSC CGIs (downloaded from the UCSC genome browser [3]. The black-gray-white pie charts show the composition of CG methylation. Low-methylated CGs (10% to 50% methylated, in gray) are enriched in tissue-specific HMRs while unmethylated CGs (<10% methylated, in white) are enriched in common HMRs.

Figure S7: Heatmap of CG methylation for the different groups of keratinocyte HMRs in different cells. Heatmap of CG methylation for the different groups of keratinocyte HMRs (S1, C1-C5) in fibroblasts and keratinocytes along with two published methylomes for embryonic stem (ES) and neuronal progenitor (NP) cells [4]. The common HMRs are unmethylated (red) in all four methylomes.

Figure S8: Comparison of HMRs in fibroblasts, keratinocytes, embryonic stem cells (ESC) and neuronal progenitor cells (NP). (**a**) Venn diagram of overlapping of HMRs in fibroblasts (49,233), keratinocytes (71,495), ESC (40,582) and NP (30,151) cells. (**b**) Tabular representation of the overlapping HMRs in four cell types.

Figure S9: HMRs are the conserved regions with high GC content as well as CpG density. (a-e) Average #CG/Kb (**a**), GC content (**b**), Observed/Expected CpG ratio (**c**), Observed/Expected TpG ratio (**d**) and PhastCons conservation scores (**e**) for five groups: 16,000 USCS CGI, common HMRs (C1), tissue-specific fibroblast and keratinocyte HMRs (S1), and randomly selected regions from the mouse genome as control.

Figure S10: SINE elements are depleted inside the HMRs, but enriched in the surrounding regions; however, both LTR and LINE elements are depleted in both inside and at the surrounding regions. (**a**) Heatmap of distribution of SINE in keratinocyte-specific HMRs. (**b**) Summary plot of SINE distribution in the five groups shown in Figure S9. (**c**) Heatmap of

distribution of LINE and LTR in keratinocyte-specific HMRs. (**d-e**) Summary plot of LINE (**d**) and LTR (**e**) distribution in the five groups shown in **Figure S9**.

Figure S11: Comparison of mRNA expression between fibroblasts and keratinocytes. (**a-b**) Comparison of mRNA expression (RPKM from RNA-seq) between biological replicates in (**a**) fibroblasts and (**b**) keratinocytes. (**c**) Pearson correlation of fibroblasts and keratinocytes RNA-seq data. (**d**) Comparison of Fb. and Ker. mRNA expression as obtained from RNA-seq and expressed in reads per kilobase per million mapped reads (RPKM). The dark blue are fibroblast-specific genes and the dark red are keratinocyte-specific genes. (**e-f**) Gene ontology (GO) analysis showed enrichment of tissue-specific genes expression for (**e**) keratinocytes and (**f**) fibroblasts.

Figure S12: Comparison of mRNA expression between fibroblasts and keratinocytes. (**a-h**) Scatter plots of dermal fibroblasts and keratinocyte gene expression (RPKM) that have (**a**) identical boundary HMRs, (**b**) extended HMR in dermal fibroblasts and (**c**) extended HMR in keratinocytes (**d**) C3 class, (**e**) C4L/S class, (**f**) C4S/L class, (**g**) C5L/S class, (**h**) C5S/L class within 1-kbp of TSS.

Figure S13: UCSC genome browser screen shots as examples for different types of HMRs. (a) Fibroblast-specific TS-HMR (S1) leading to isoforms-level differential expression of *Arap1* in primary dermal fibroblasts. (**b**) Extension of both sides of HMR in promoter of *Col1a2* in fibroblasts leading to gene expression. (**c**) Tissue-specific differential exonic CGI methylation for *Hoxc13* with low methylation in fibroblast with no expression, and high methylation in keratinocytes with high expression. (**d**) Differential exonic CGI methylation is the extended of HMRs towards the gene body leading to *Hya12* high expressed in keratinocytes.

Figure S14: UCSC genome browser screen shots as examples for tissue specific genes expression with TS-HMRs near the TSS of *Krt1*, *Krt5* and *NNMT1*.

Figure S15: UCSC genome browser screen shots as examples for extended HMRs leading to enhanced gene expression of *Emilin1*, *Trp53il1* and *Wnt3*.

Figure S16: Correlation between the methylation difference and gene expression changes. (**a**) Correlation between the methylation difference and gene expression fold changes in fibroblasts

and keratinocytes. (**b**) Comparison of mRNA expression (RPKM) between fibroblasts and keratinocytes for gene with differential methylated CGIs at exons. (**c-d**) Comparison of mRNA expression (RPKM) between genes with CGIs and without CGIs in (**c**) fibroblasts and (**d**) keratinocytes.

Figure S17: Exons with methylated conserved CGs are highly expressed. (**a-b**) Boxplot of the gene expression (RPKM) for the unmethylated (0 to10%) and methylated (90% to 100%) Exons with different conservation score in (**a**) fibroblasts and in (**b**) keratinocytes.

Figure S18: Exons with methylated conserved CGs are highly expressed. (**a-b**) Boxplot of the gene expression (RPKM) for genes with different methylation status of exons with different conservation score in (**a**) fibroblasts and in (**b**) keratinocytes. The methylation status of the exons is classified into ten groups.

Figure S19: RNA-seq signal at the *Fat1* locus. RNA-seq reads are shown at the annotated exons, whereas at approximately 15 kbp upstream, similar RNA-seq signals indicate probable first exon of *Fat1*.

Figure S20: Transcription factor binding sites (TFBS) in HMRs. (**a**) Comparison of enriched TFBS motifs in the fibroblasts and keratinocyte-specific HMRs (S1). (**b**) Comparison of enriched TFBS motifs in keratinocyte specific HMRs and common HMRs (keratinocytes and fibroblasts) in CGIs. (**c**) Comparison of enriched TFBS motifs in fibroblast specific HMRs and common HMRs (keratinocytes and fibroblasts) in CGIs. (**d**) Comparison of enriched TFBS motifs in common HMRs (keratinocytes and fibroblasts) not in CGIs and those in CGIs.

Figure S21: Comparison of enriched TFBS motifs in the fibroblasts and keratinocyte HMRs. (**a**) mRNA expression (expressed as RPKM) of some differentially and commonly enriched transcription factors in dermal fibroblasts and keratinocytes. (**b-c**) Comparison of enriched TFBS motifs in the specific HMRs (S1) and partial specific HMR (pS, the extending regions of common HMRs) in keratinocytes (**b**) and fibroblasts (**c**). (**d-e**) Comparison of enriched TFBS motifs in the specific HMRs (S1) and the common HMRs (Cs) in keratinocytes (**d**) and fibroblasts (**e**). (**f-g**) Comparison of enriched TFBS motifs in partial specific HMR (pS, the extending regions of common HMRs) and the common HMRs (Cs) in keratinocytes (**f**) and

fibroblasts (**g**). (**h**) Heatmap of top 50 weighted motifs in the fibroblasts and keratinocyte specific HMRs (S1).

Figure S22: Binding motifs in C/EBPβ and CTCF ChIP-seq peaks. (**a**) Binding motifs for C/EBPβ ChIP-seq peaks at common C/EBPβ peaks (i), fibroblast-specific (ii) and keratinocyte-specific (iii) peaks. (**b**) Binding motifs for CTCF ChIP-seq peaks at common C/EBPβ peaks (i), fibroblast-specific (ii) and keratinocyte-specific (iii) peaks.

References:

1. Illingworth RS, Gruenewald-Schneider U, Webb S, Kerr AR, James KD, Turner DJ, Smith C, Harrison DJ, Andrews R, Bird AP: **Orphan CpG islands identify numerous conserved promoters in the mammalian genome.** *PLoS Genet* 2010, **6:**e1001134.

2. Ponger L, Mouchiroud D: **CpGProD: identifying CpG islands associated with transcription start sites in large genomic mammalian sequences.** *Bioinformatics* 2002, **18:**631–633.

3. Gardiner-Garden M, Frommer M: **CpG islands in vertebrate genomes.** *J Mol Biol* 1987, **196:**261–282.

4. Stadler MB, Murr R, Burger L, Ivanek R, Lienert F, Scholer A, van Nimwegen E, Wirbelauer C, Oakeley EJ, Gaidatzis D, Tiwari VK, Schübeler D: **DNA-binding factors shape the mouse methylome at distal regulatory regions.** *Nature* 2011, **480:**490–495.

Figure S1

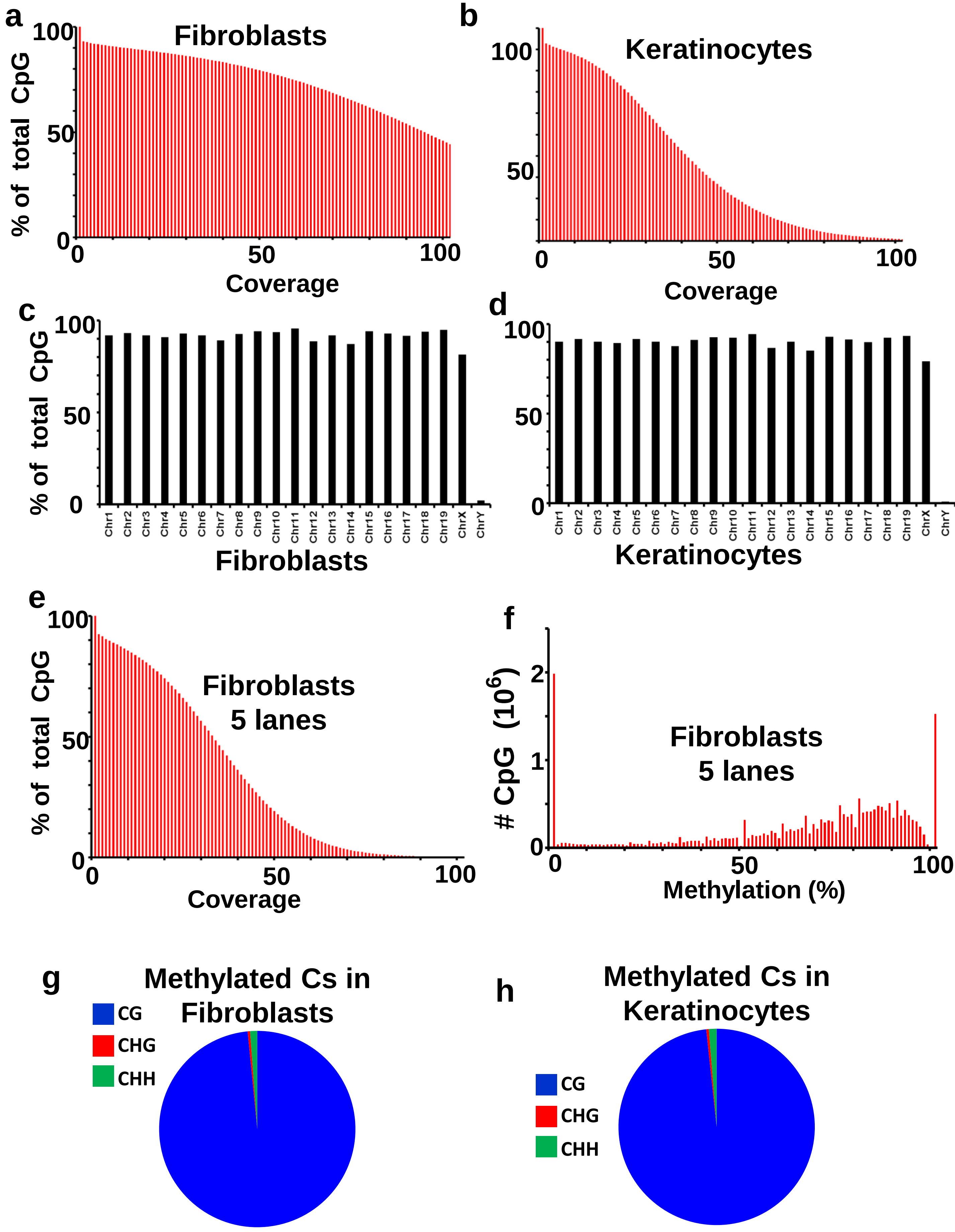
a
Fibroblasts
% of total CpG
100
50
0
0
50
100
Coverage
b
Keratinocytes
100
50
0
50
100
Coverage
c
% of total CpG
100
50
0
Chr1
Chr2
Chr3
Chr4
Chr5
Chr6
Chr7
Chr8
Chr9
Chr10
Chr11
Chr12
Chr13
Chr14
Chr15
Chr16
Chr17
Chr18
Chr19
ChrX
ChrY
Fibroblasts
d
100
50
0
Keratinocytes
e
% of total CpG
Fibroblasts
5 lanes
100
50
0
0
50
100
Coverage
f
CpG (10^6)
2
1
0
Fibroblasts
5 lanes
0
50
100
Methylation (%)
g
Methylated Cs in
Fibroblasts
CG
CHG
CHH
h
Methylated Cs in
Keratinocytes
CG
CHG
CHH

Figure S2

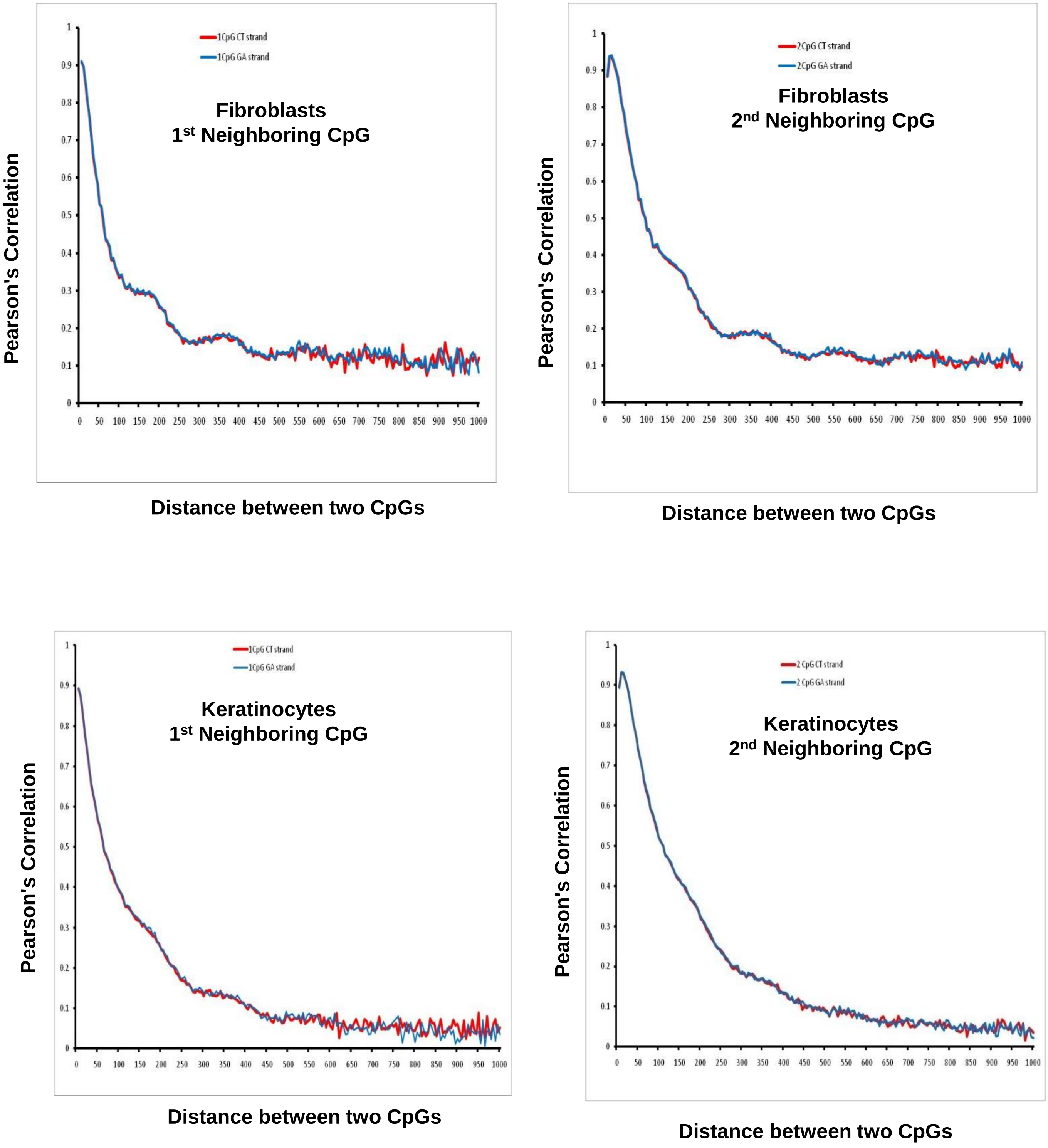
1CpG CT strand
1CpG GA strand
Fibroblasts
1st Neighboring CpG
Pearson's Correlation
Distance between two CpGs
2CpG CT strand
2CpG GA strand
Fibroblasts
2nd Neighboring CpG
Pearson's Correlation
Distance between two CpGs
1CpG CT strand
1CpG GA strand
Keratinocytes
1st Neighboring CpG
Pearson's Correlation
Distance between two CpGs
2 CpG CT strand
2 CpG GA strand
Keratinocytes
2nd Neighboring CpG
Pearson's Correlation
Distance between two CpGs

Figure S3

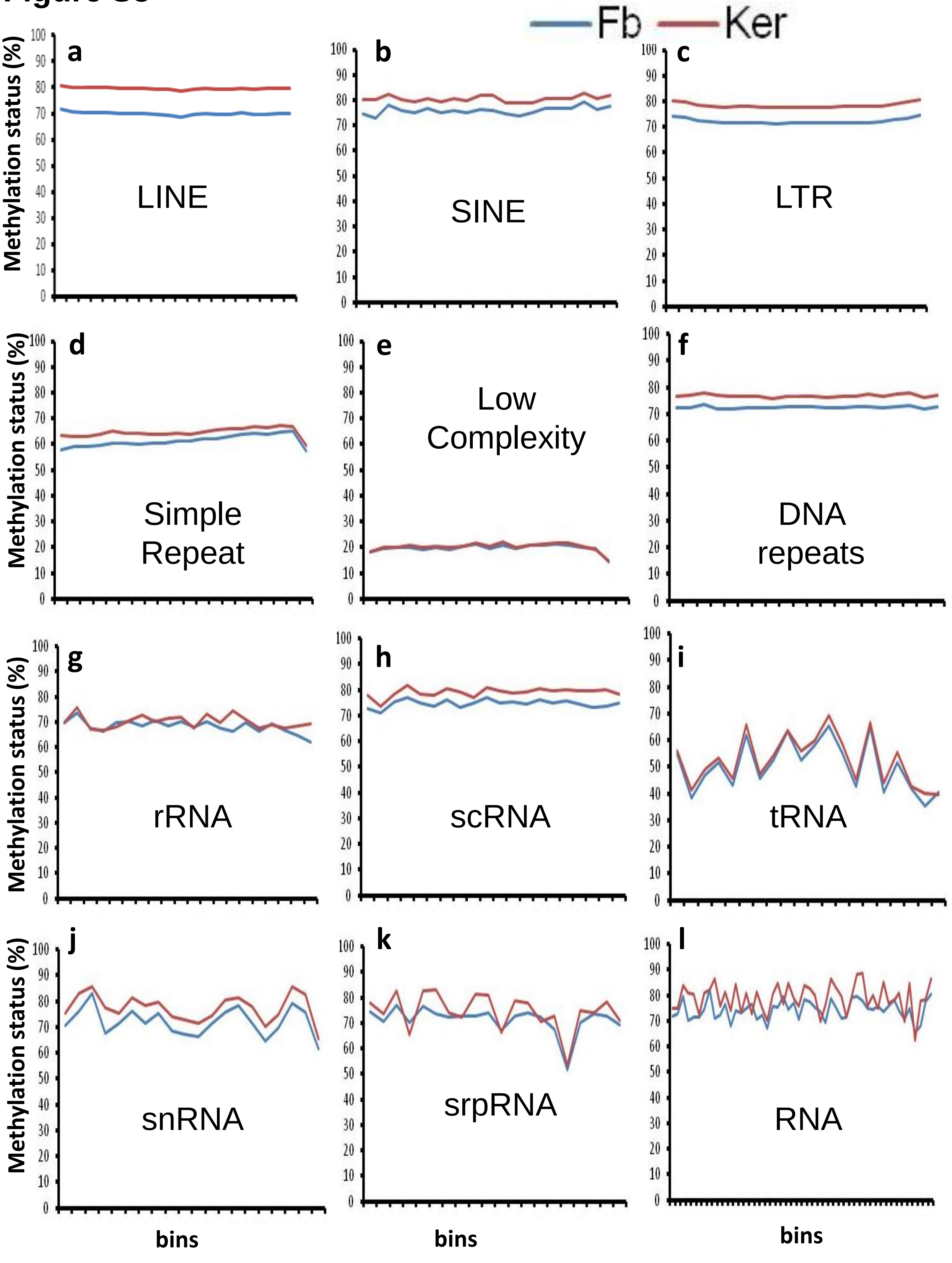

Fb
Ker
Methylation status (%)
a
LINE
b
SINE
c
LTR
d
Simple Repeat
e
Low Complexity
f
DNA repeats
g
rRNA
h
scRNA
i
tRNA
j
snRNA
k
srpRNA
l
RNA
0
10
20
30
40
50
60
70
80
90
100
bins

Figure S4

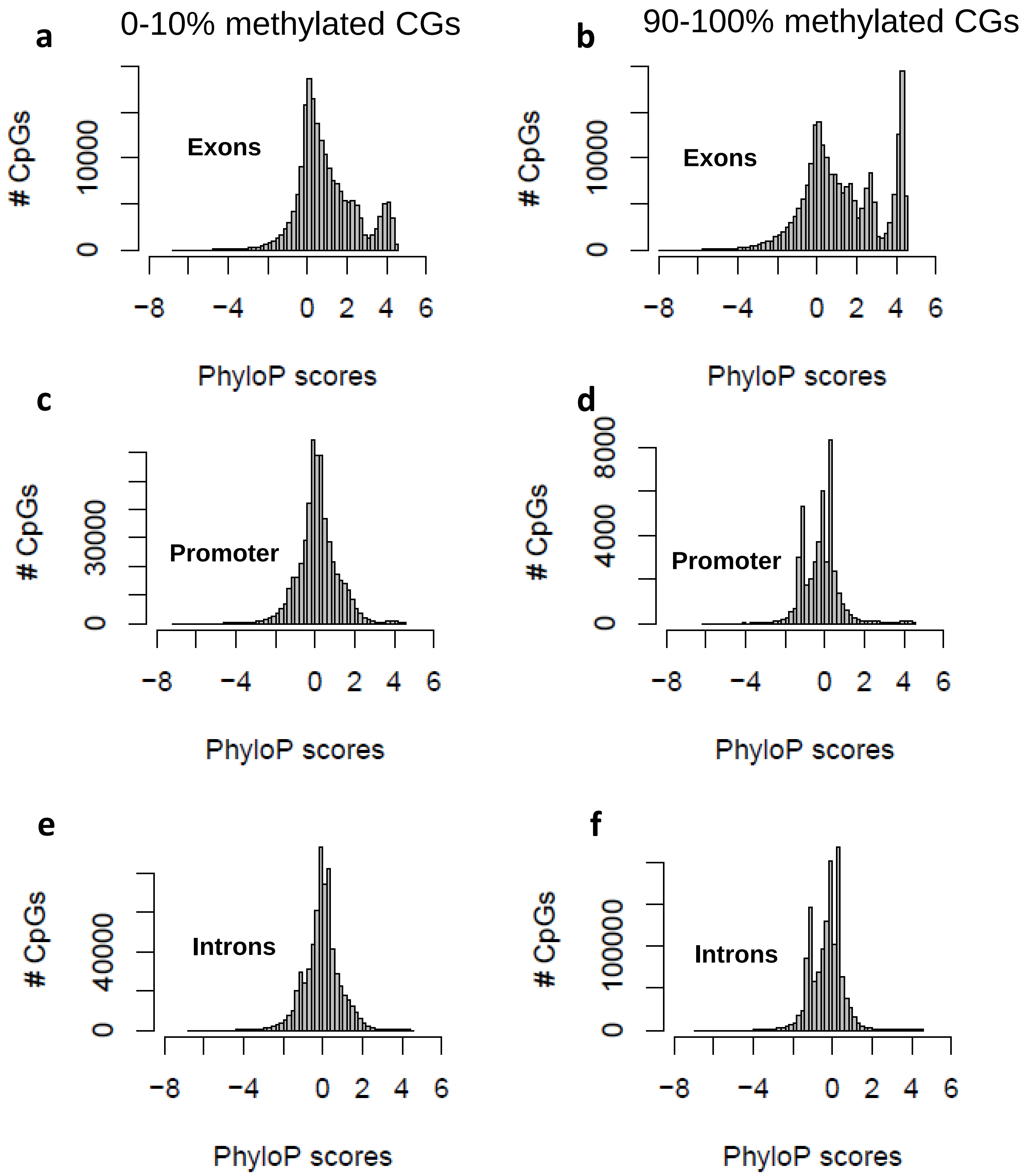
0-10% methylated CGs
90-100% methylated CGs
a
b
c
d
e
f
Exons
Exons
Promoter
Promoter
Introns
Introns
CpGs
0
10000
30000
40000
4000
8000
100000
−8
−4
0
2
4
6
PhyloP scores

Figure S5

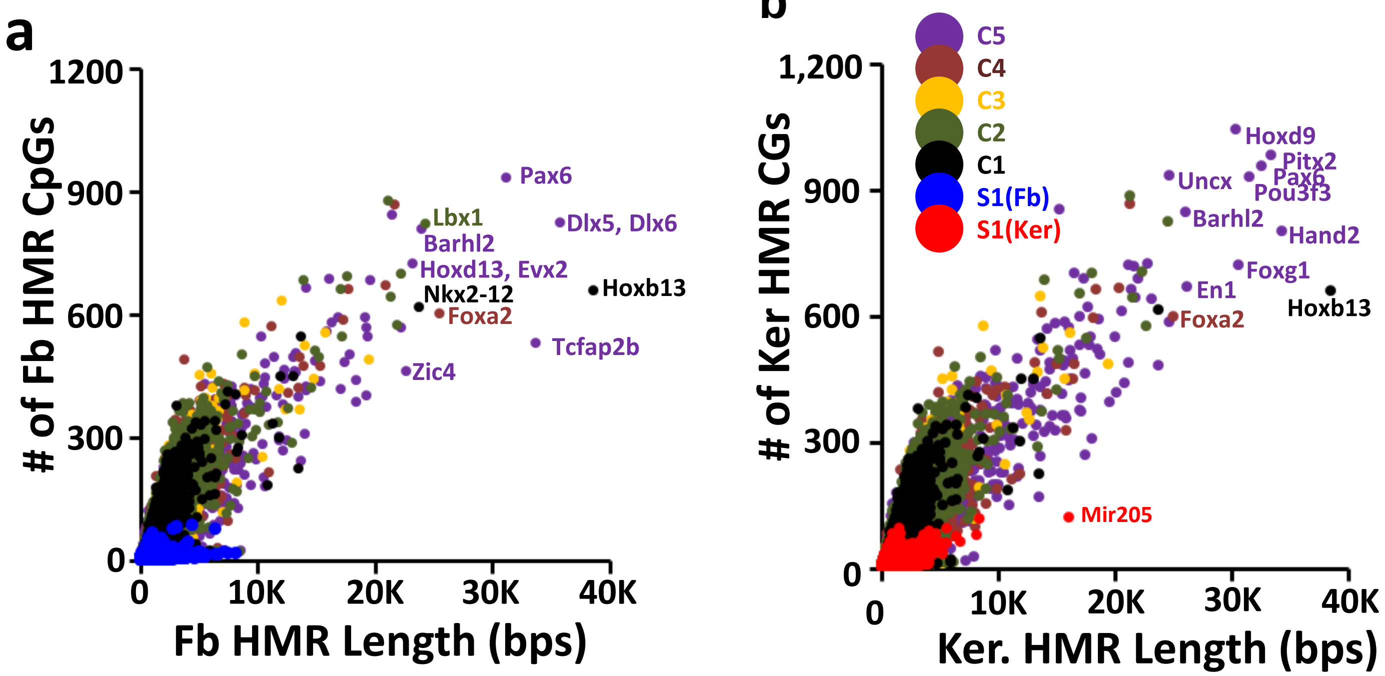

c. C1: Largest common HMR covered *Hoxb13* with no expression in Fb. and Ker.

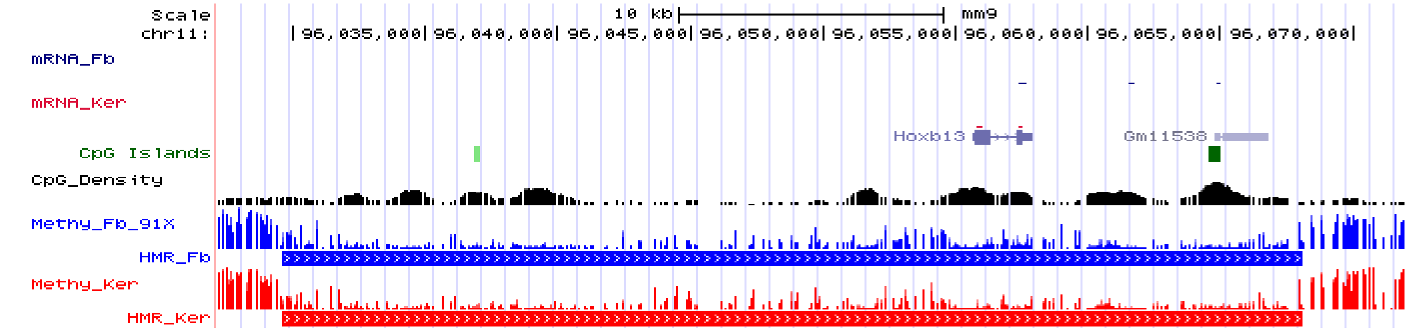

d. S1: Largest Ker-specific HMR covered *Mir205* locus with expression only in Ker.

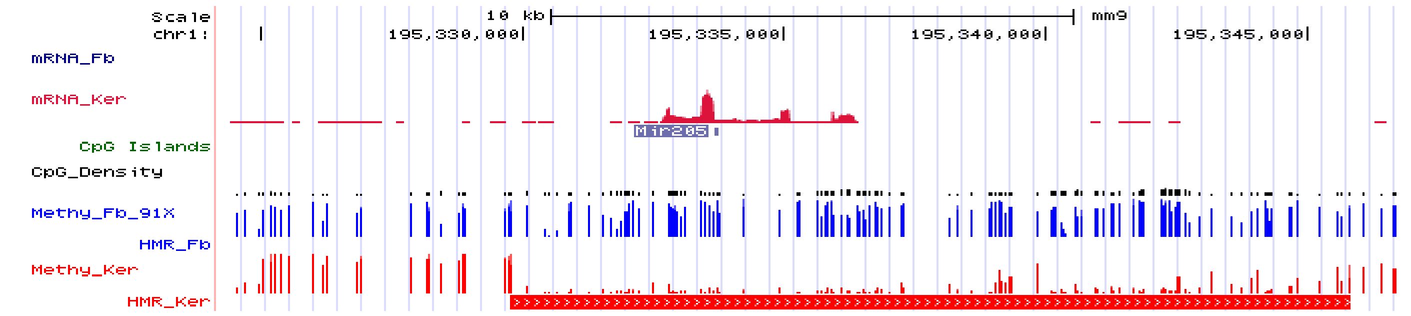

e. S1: Largest Fb-specific HMR located in the *Skint6* locus without expression.

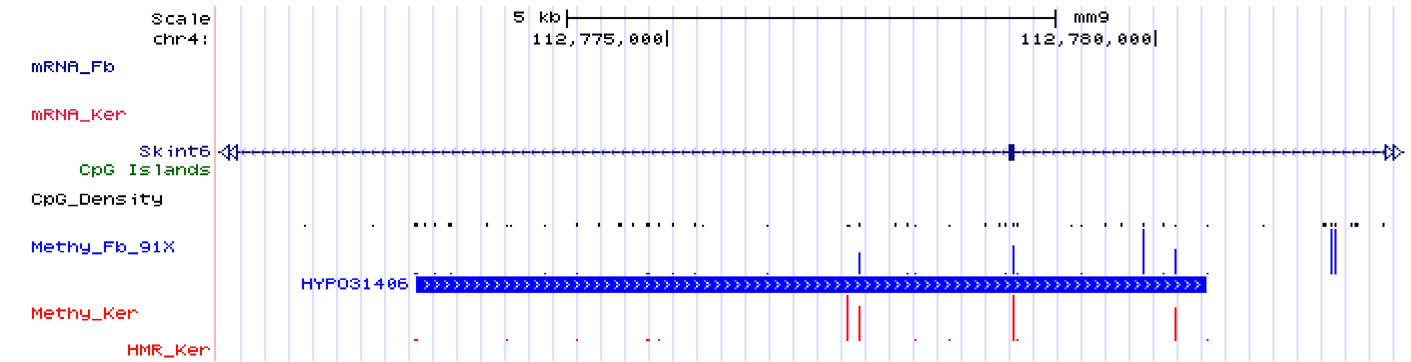

Figure S6

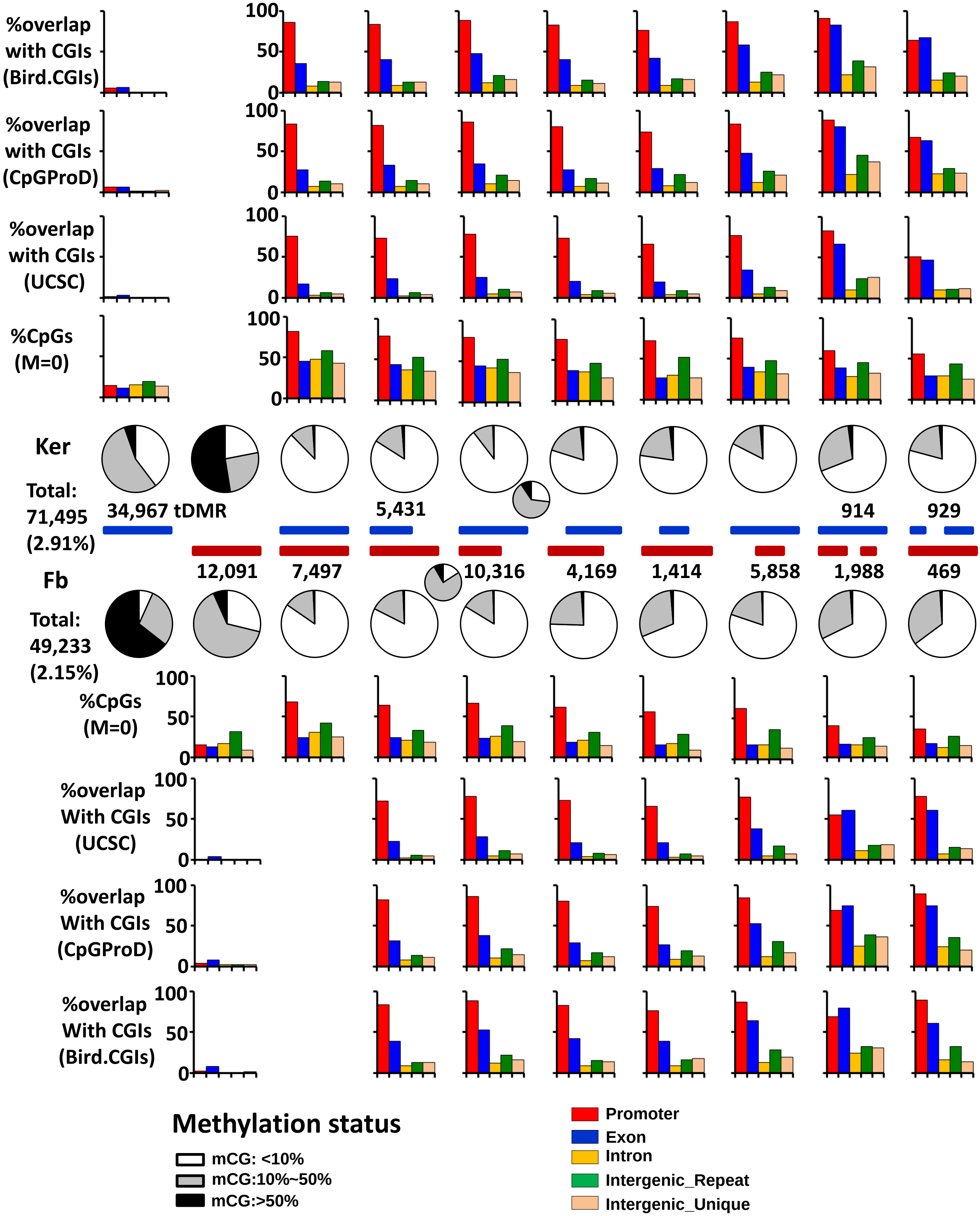

%overlap with CGIs (Bird.CGIs)
%overlap with CGIs (CpGProD)
%overlap with CGIs (UCSC)
%CpGs (M=0)
100
50
0
Ker
Total: 71,495 (2.91%)
34,967 tDMR
5,431
914
929
12,091
7,497
10,316
4,169
1,414
5,858
1,988
469
Fb
Total: 49,233 (2.15%)
%CpGs (M=0)
%overlap With CGIs (UCSC)
%overlap With CGIs (CpGProD)
%overlap With CGIs (Bird.CGIs)
Methylation status
mCG: <10%
mCG:10%~50%
mCG:>50%
Promoter
Exon
Intron
Intergenic_Repeat
Intergenic_Unique

Figure S7

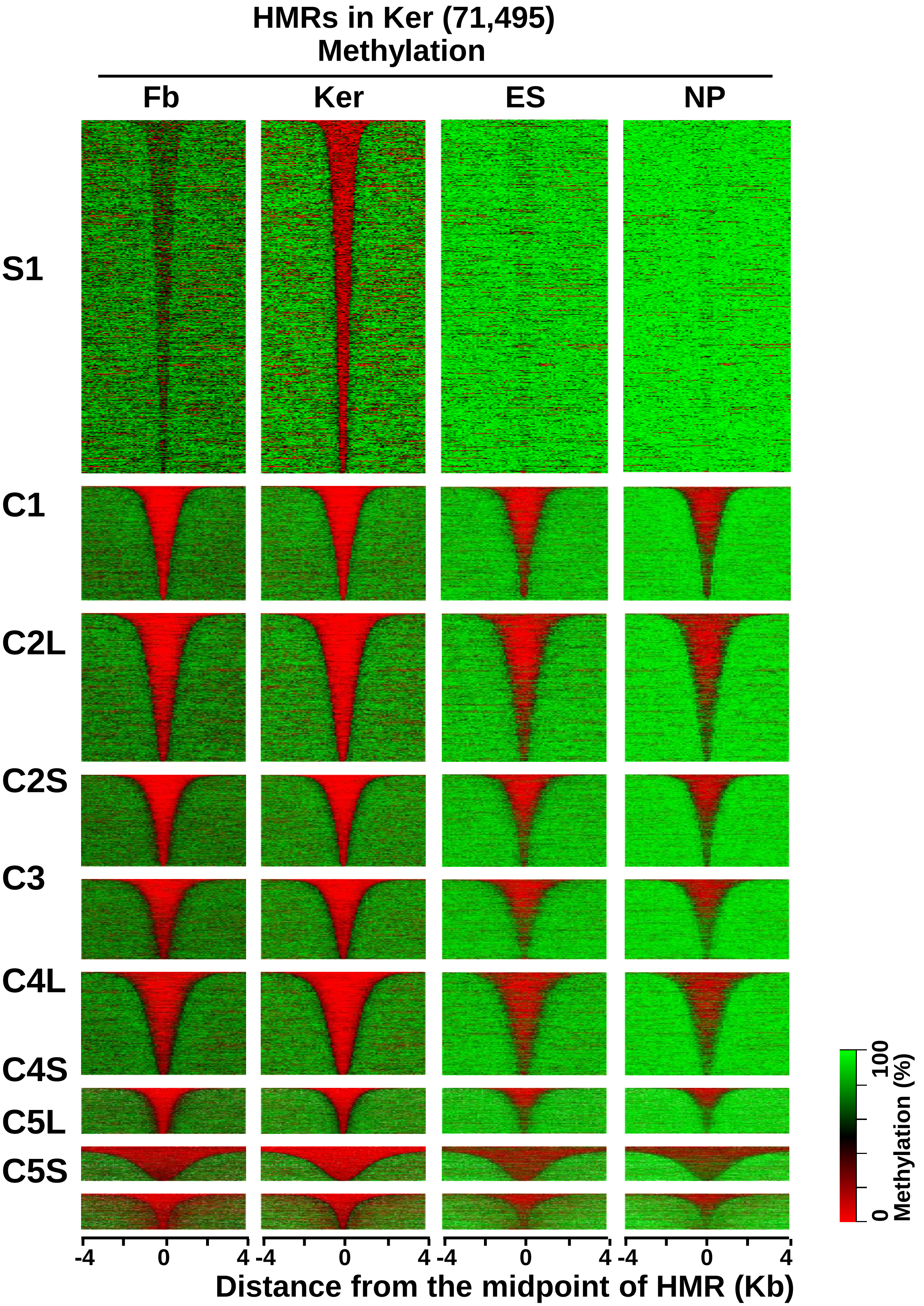
HMRs in Ker (71,495)
Methylation
Fb
Ker
ES
NP
S1
C1
C2L
C2S
C3
C4L
C4S
C5L
C5S
-4
0
4
Distance from the midpoint of HMR (Kb)
100
0
Methylation (%)

Figure S8

a

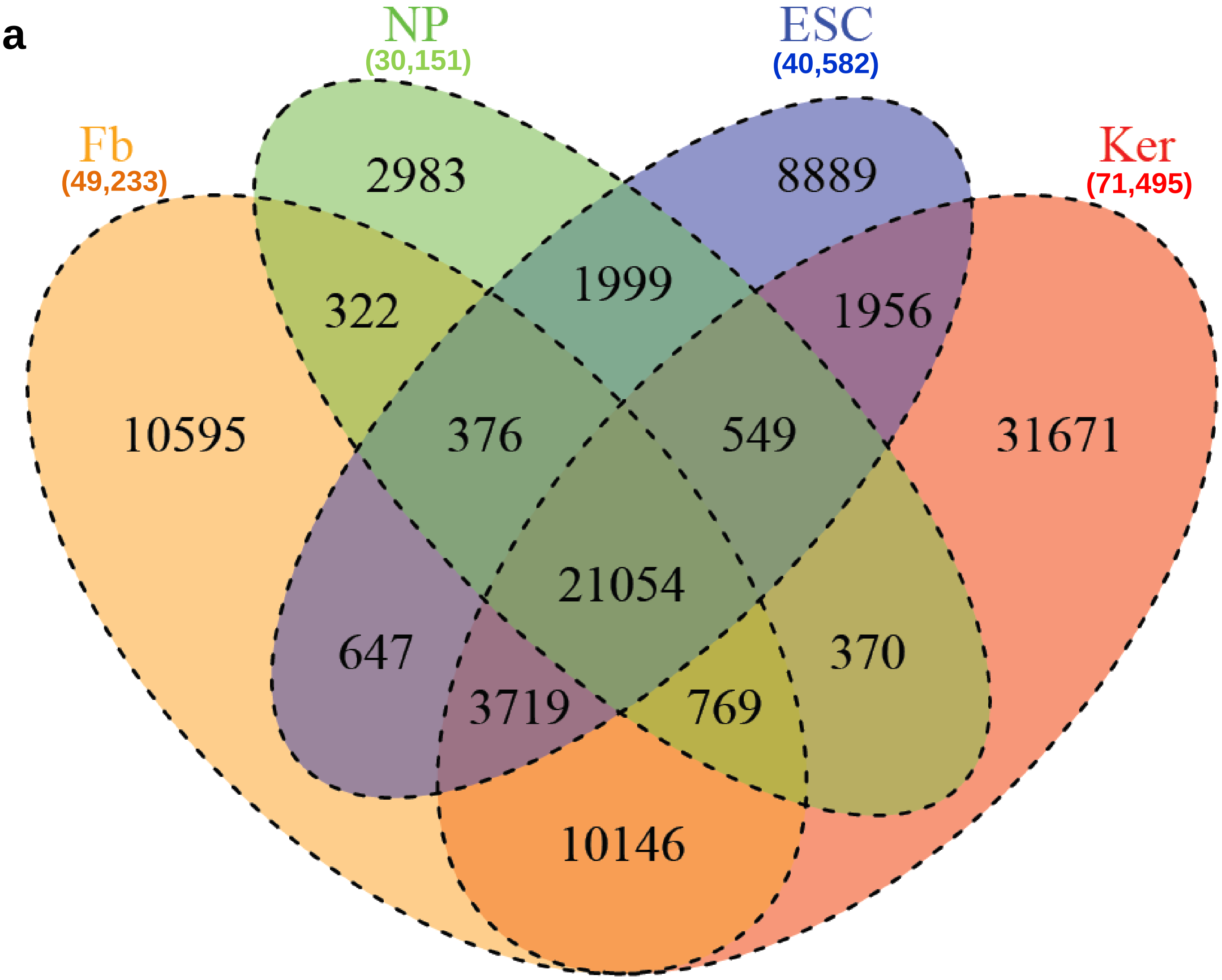

b

	Fb	Ker	NP	ESC	All 4 - X	All 4
**Fb**	10,595				549	21,054
**Ker**	10,146	31,671			376	
**NP**	322	370	2,983		3,719	
**ESC**	647	1,956	1,999	8,889	769	

Figure S9

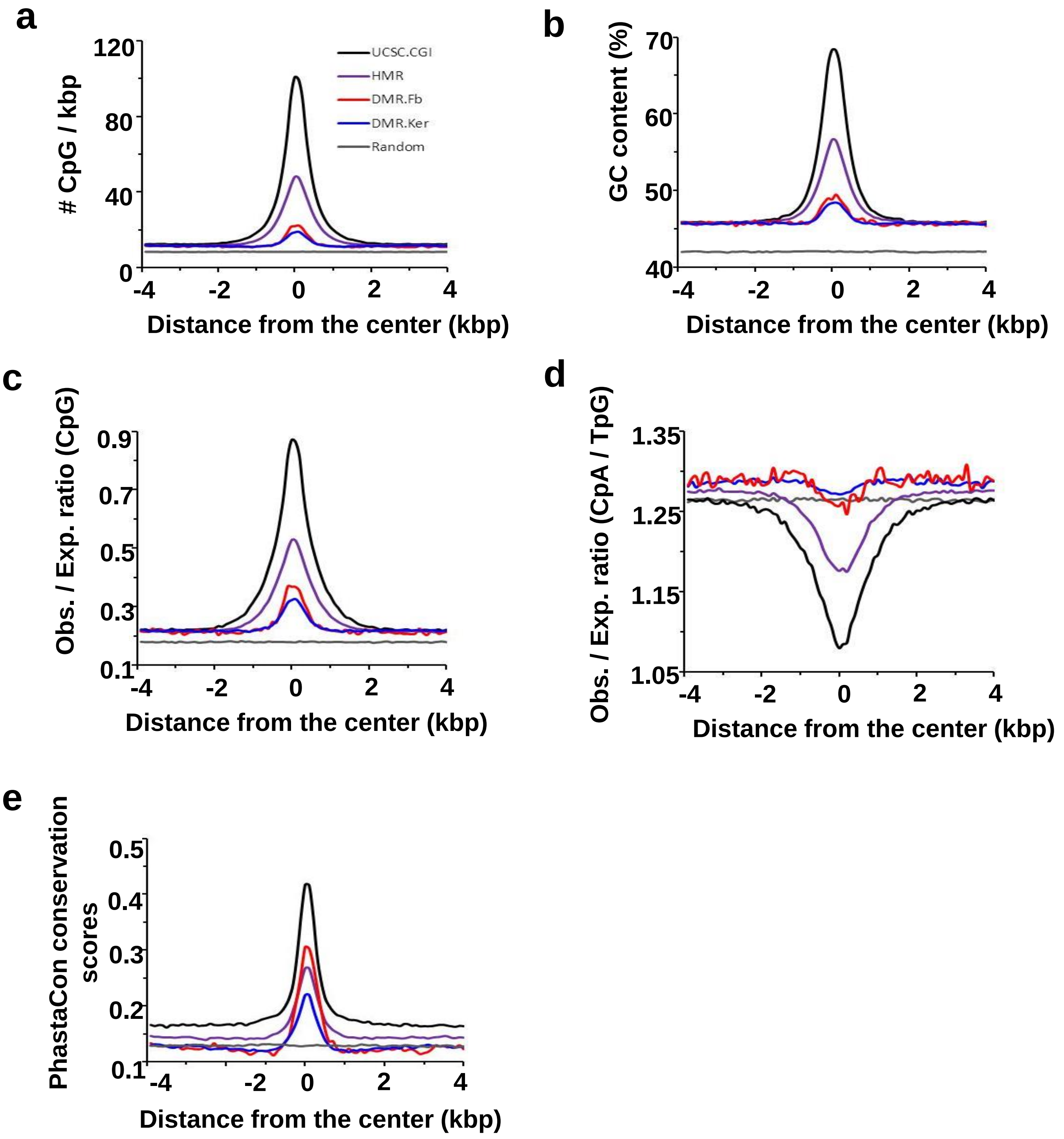
a
b
c
d
e
UCSC.CGI
HMR
DMR.Fb
DMR.Ker
Random
CpG / kbp
GC content (%)
Obs. / Exp. ratio (CpG)
Obs. / Exp. ratio (CpA / TpG)
PhastaCon conservation scores
Distance from the center (kbp)

Figure S10

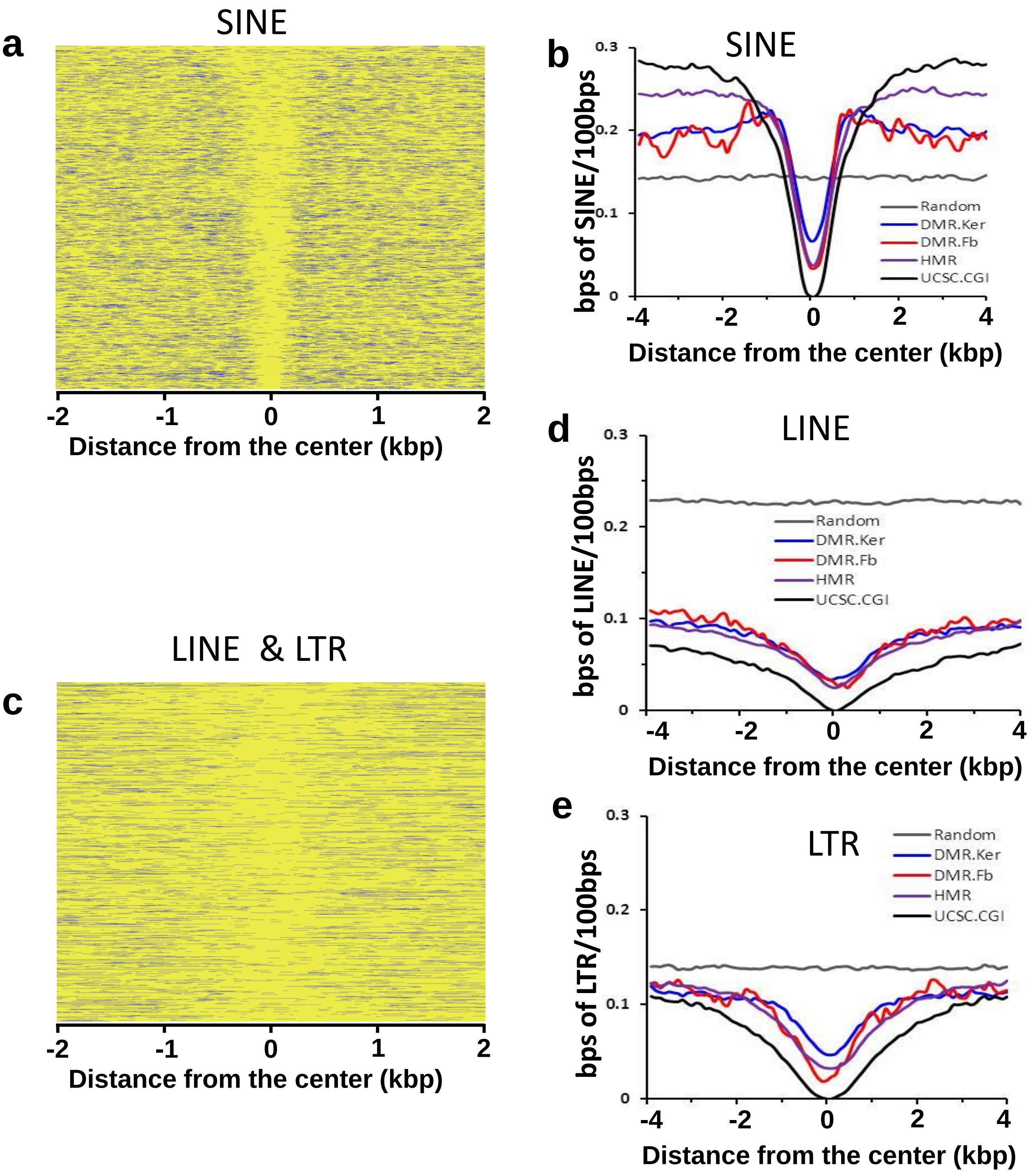

a
SINE
-2
-1
0
1
2
Distance from the center (kbp)
b
SINE
bps of SINE/100bps
0.3
0.2
0.1
0
Random
DMR.Ker
DMR.Fb
HMR
UCSC.CGI
-4
-2
0
2
4
Distance from the center (kbp)
c
LINE & LTR
-2
-1
0
1
2
Distance from the center (kbp)
d
LINE
bps of LINE/100bps
0.3
0.2
0.1
0
Random
DMR.Ker
DMR.Fb
HMR
UCSC.CGI
-4
-2
0
2
4
Distance from the center (kbp)
e
LTR
bps of LTR/100bps
0.3
0.2
0.1
0
Random
DMR.Ker
DMR.Fb
HMR
UCSC.CGI
-4
-2
0
2
4
Distance from the center (kbp)

Figure S11

a

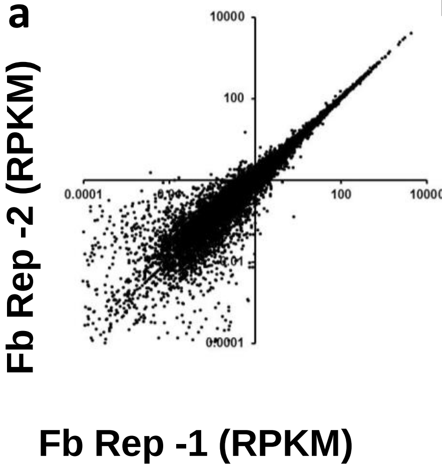

b

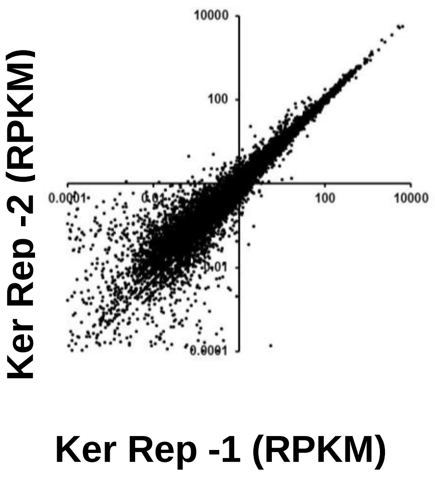

c **Pearson correlation RNA-seq data**

	Fb repl-1	Fb repl-2	Ker repl-1	Ker repl-2
Fb repl-1	1			
Fb repl-2	0.999	1		
Ker repl-1	0.190	0.196	1	
Ker repl-2	0.201	0.206	0.994	1

d

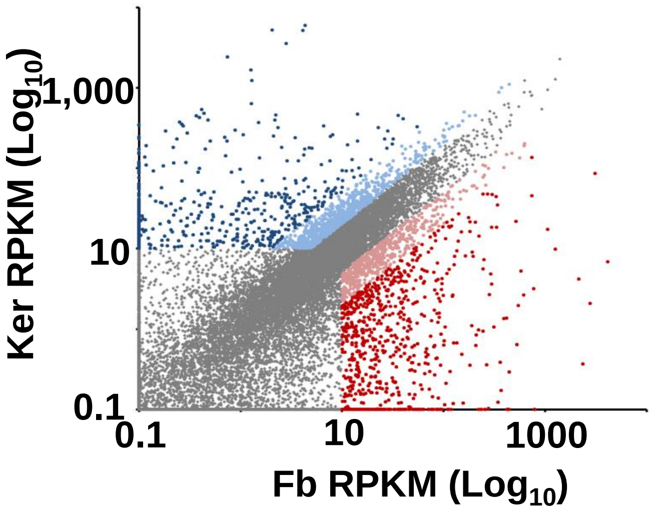

e

GO Terms for Ker	PValue	Fold Enrichment
keratinization	1.4E-16	38.70
epidermis development	4.2E-16	13.34
ectoderm development	1.5E-15	12.54
keratinocyte differentiation	8.1E-15	24.31

f

GO Terms for Fb	PValue	Fold Enrichment
cell adhesion	4.9E-18	4.36
extracellular matrix organization	7.7E-15	10.39
immunoglobulin mediated immune response	4.0E-08	9.52
response to wounding	4.8E-07	3.46

Figure S12

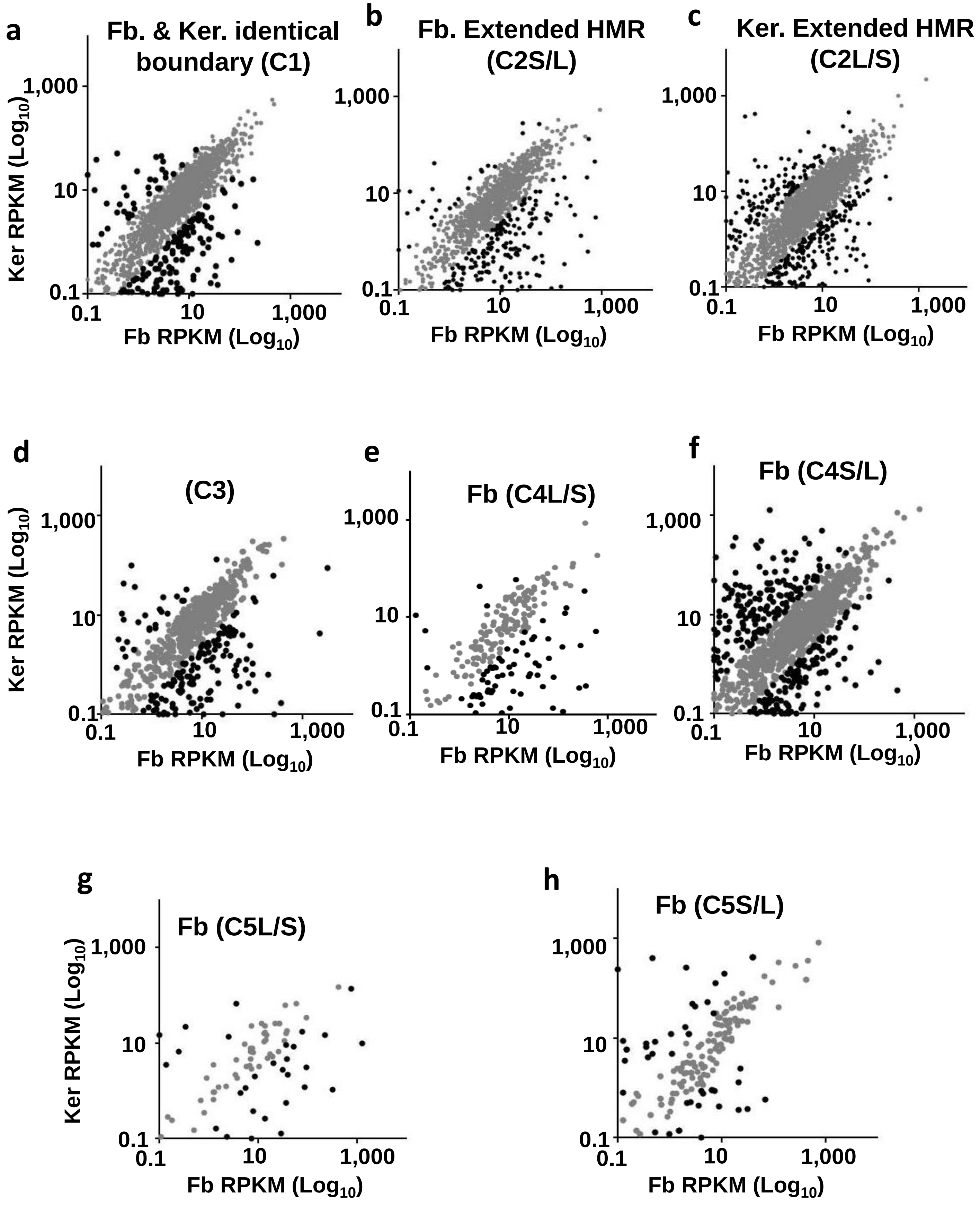
a
Fb. & Ker. identical boundary (C1)
b
Fb. Extended HMR (C2S/L)
c
Ker. Extended HMR (C2L/S)
d
(C3)
e
Fb (C4L/S)
f
Fb (C4S/L)
g
Fb (C5L/S)
h
Fb (C5S/L)
Ker RPKM (Log10)
Fb RPKM (Log10)
0.1
10
1,000

Figure S13

a Isoform level expression of *Arap1*

b C4: Extension of both sides of HMR in promoter of *Col1a2* in Fb leading to gene expression.

c Differential exonic CGI methylation in the extended HMRs towards the gene body of *HOXC13* and *Hya12*.

d

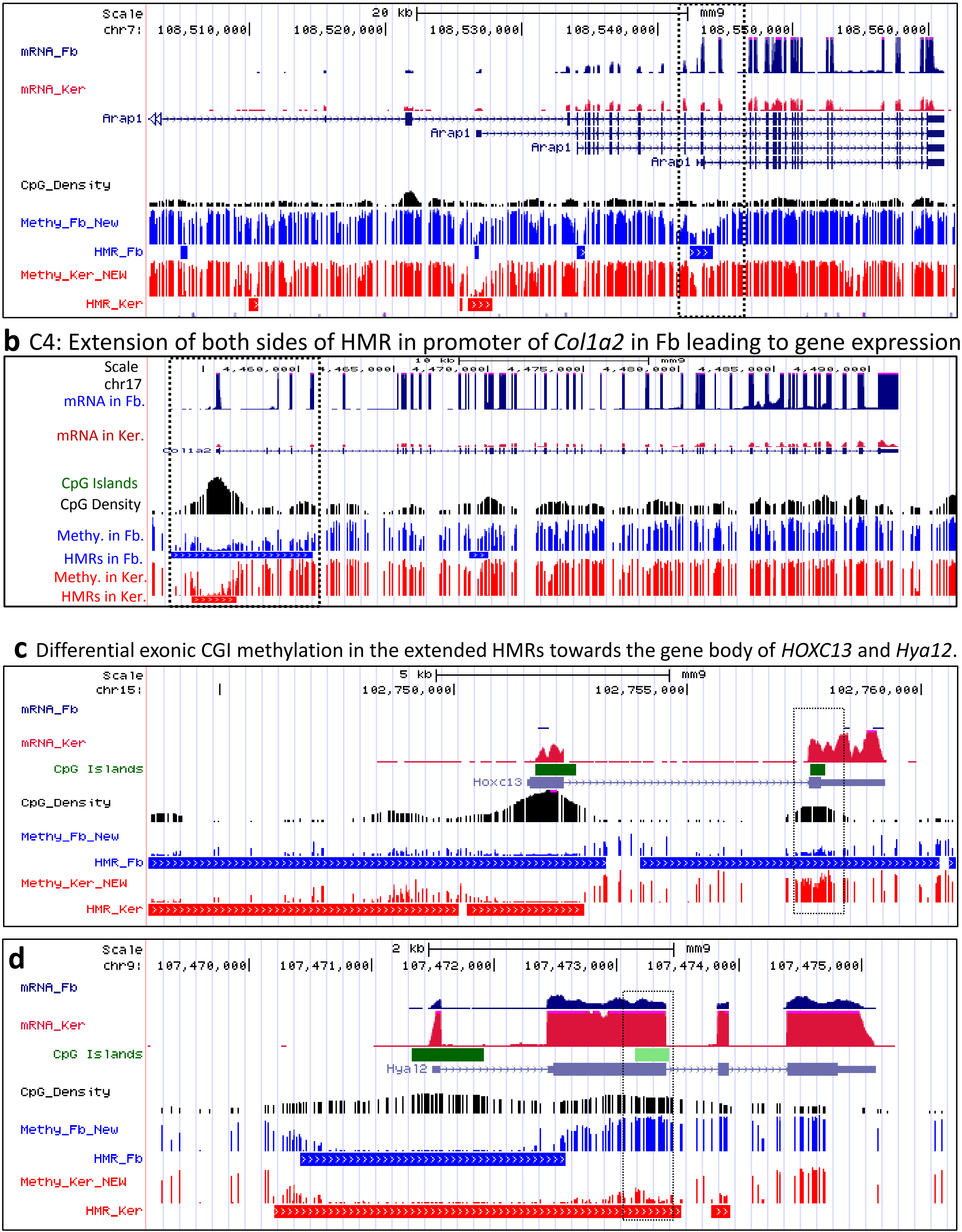

Figure S14

S1: Specific HMRs leading to *Krt1* and *Krt5* expression in Ker .

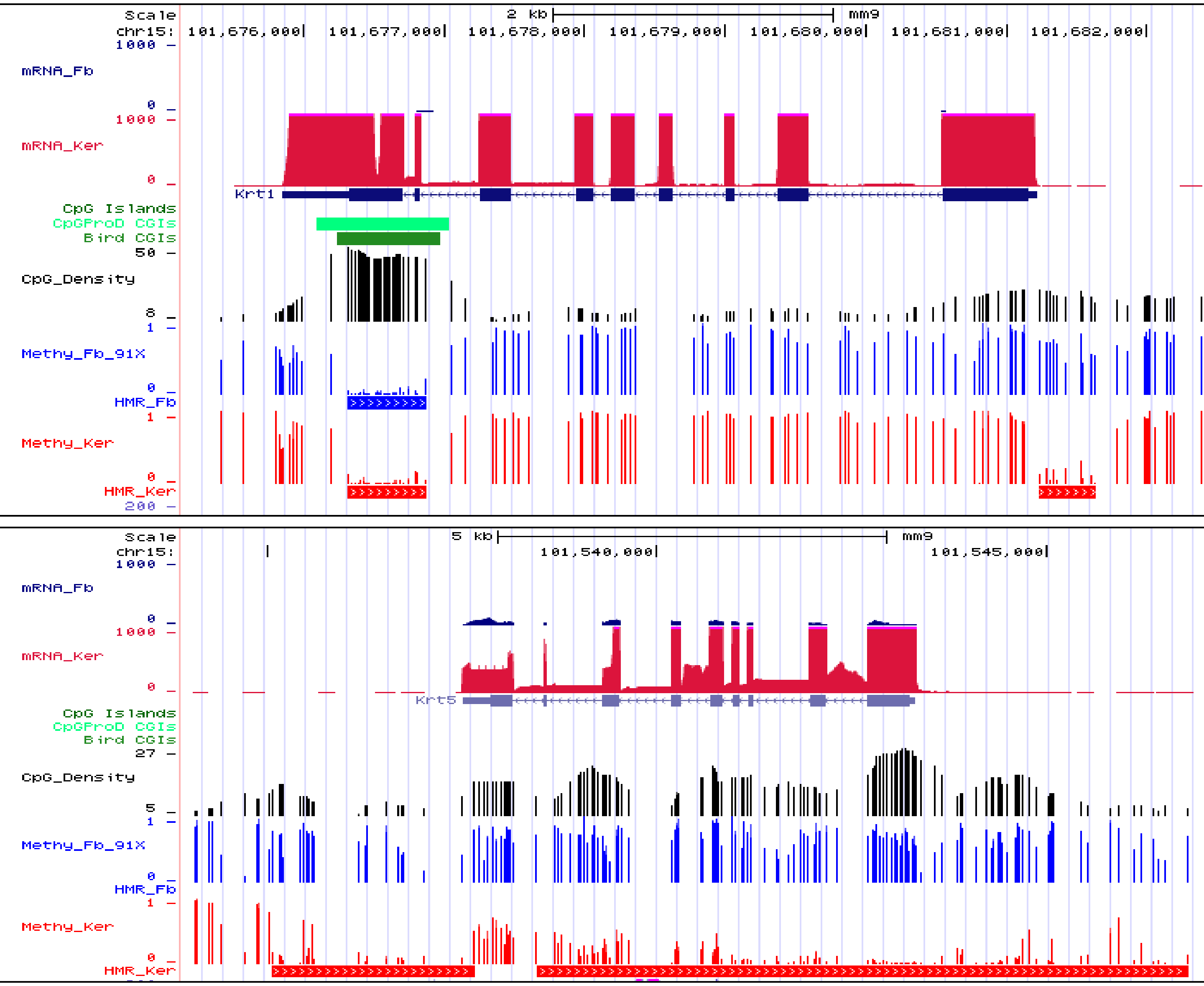

S1: Specific HMR leading to *Nnmt* expression in Fb .

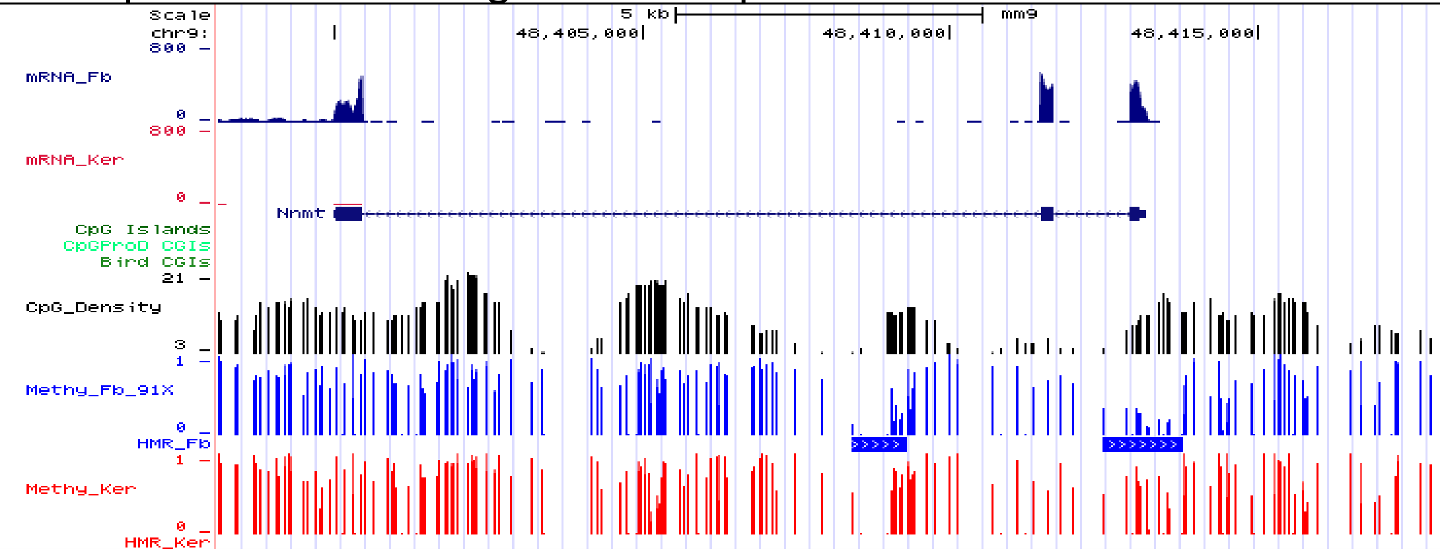

Figure S15

C2: Extension of HMR in Fb in promoter of *Emilin1* leading to high expression in Fb

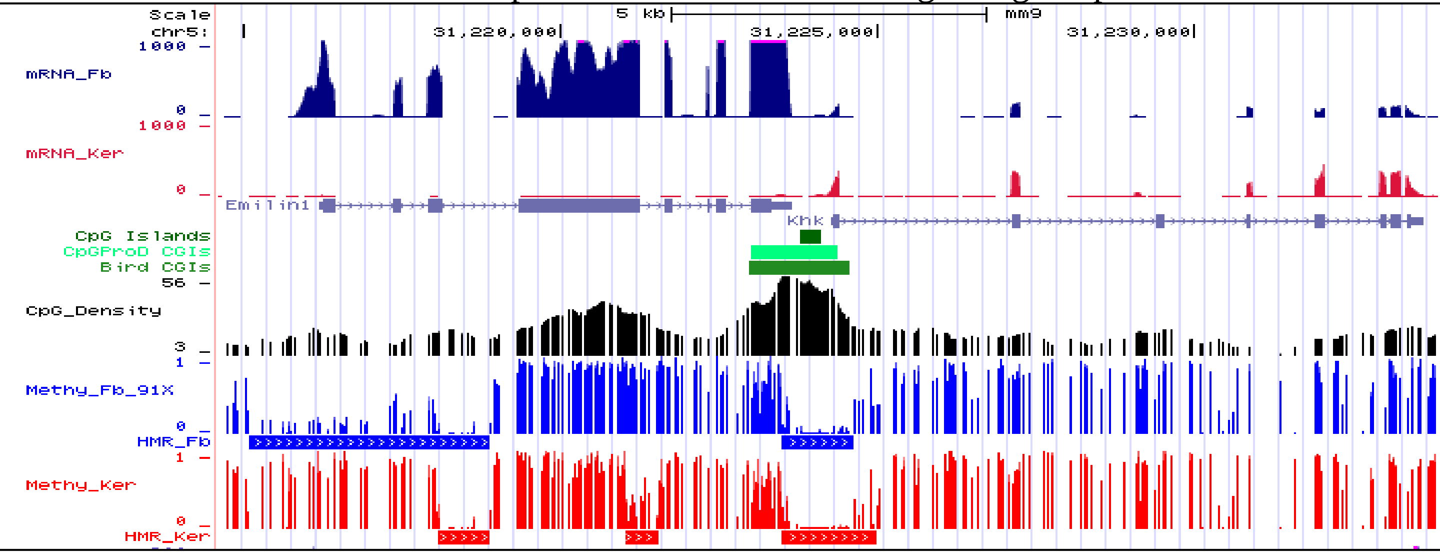

C2: Extension of HMR in Ker in promoter of *Trp53i11* leading to high expression while very low expression in Fb.

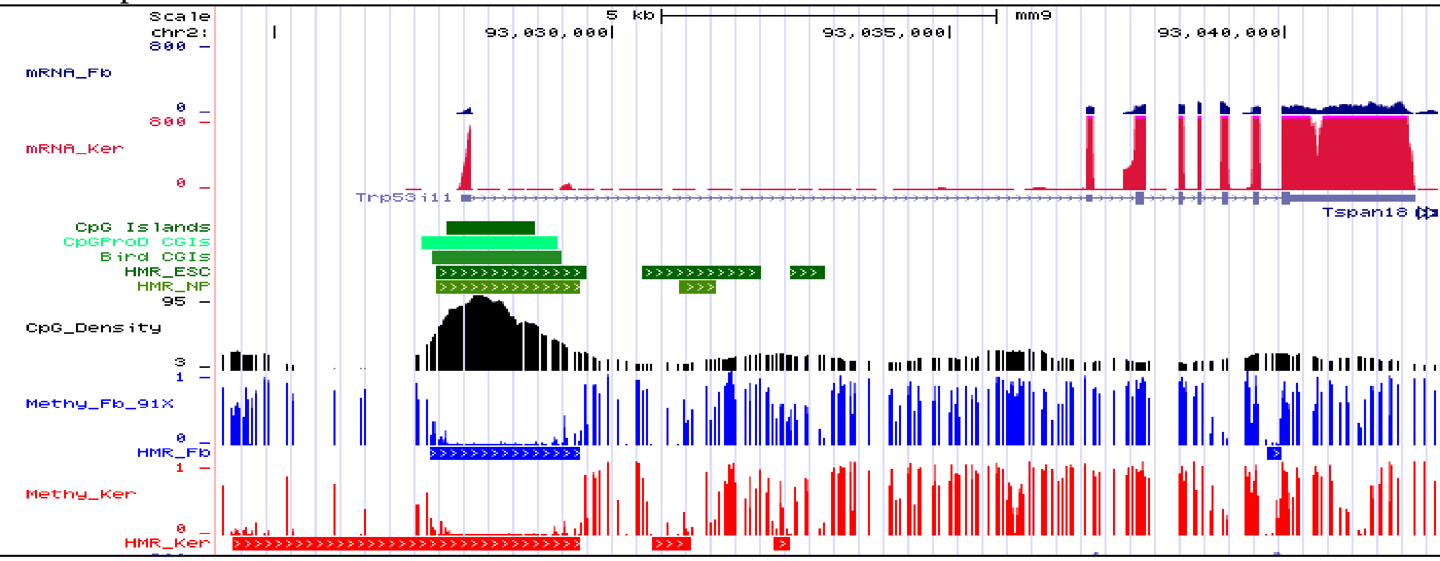

C3: Extension of HMRs towards the promoter of *Wnt3* in Ker leading to the differential gene expression.

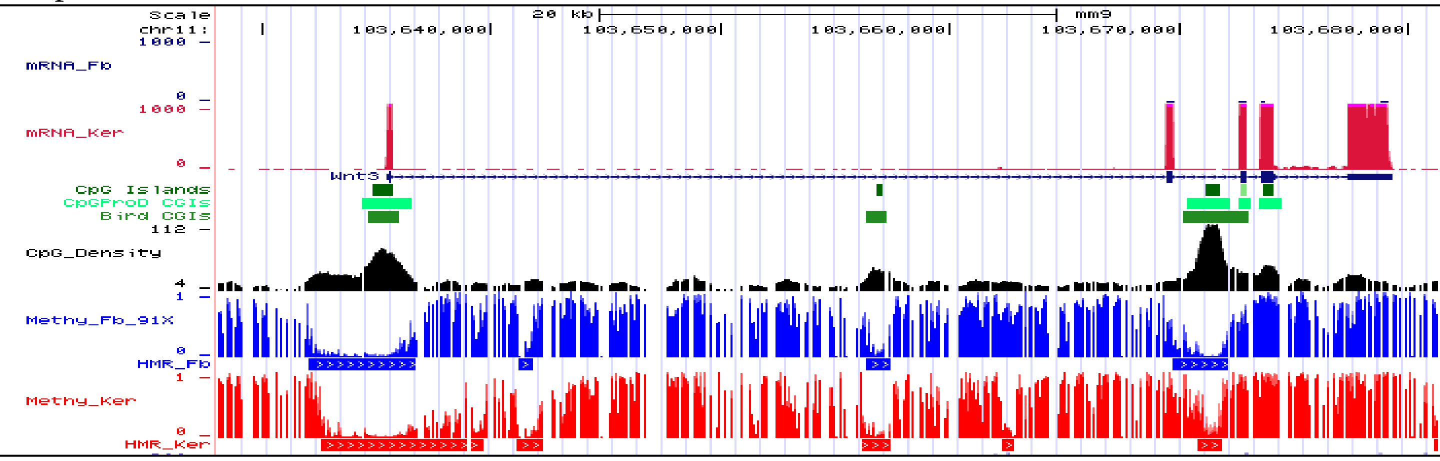

Figure S16

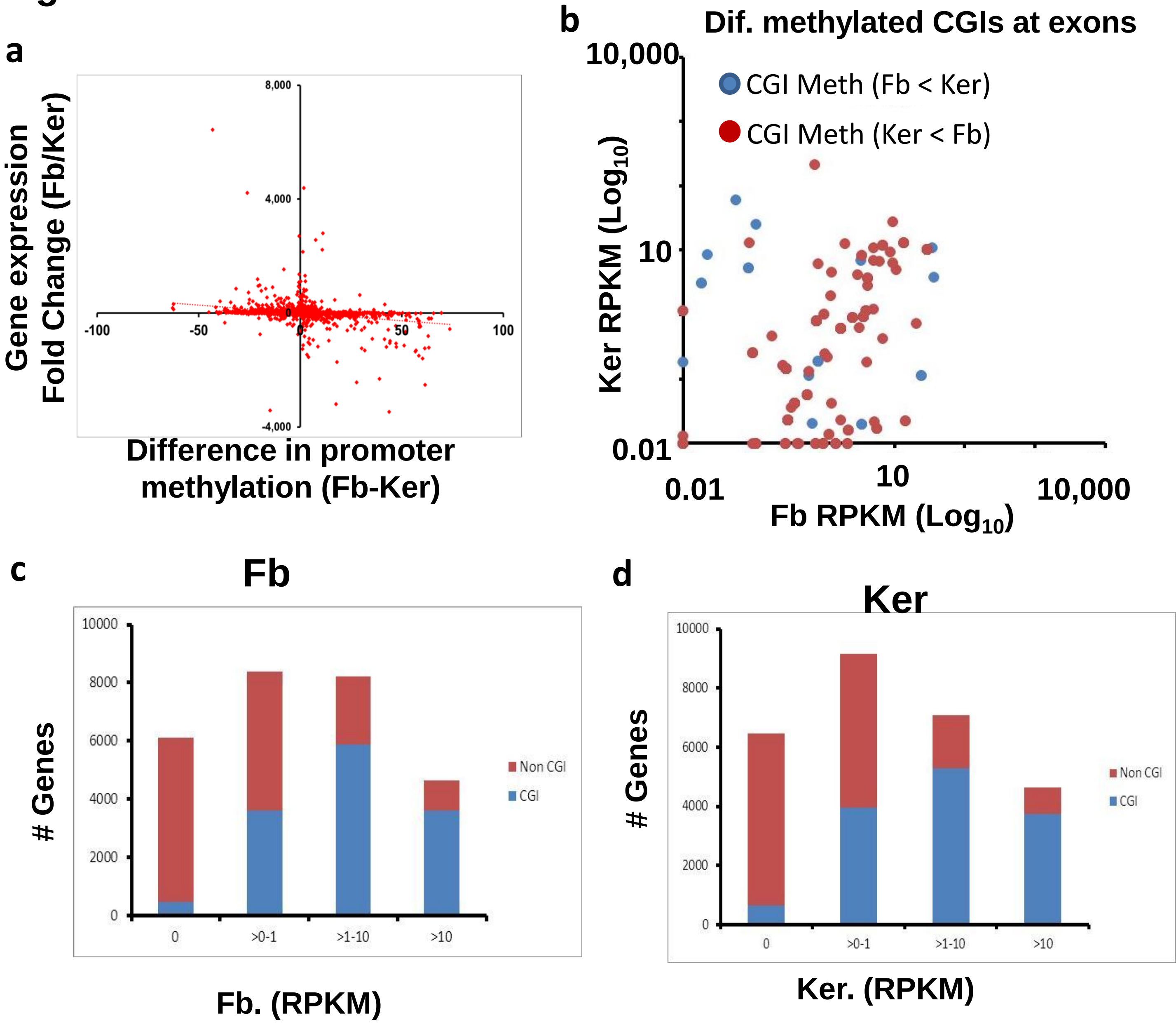
a
Gene expression Fold Change (Fb/Ker)
8,000
4,000
0
-4,000
-100
-50
0
50
100
Difference in promoter methylation (Fb-Ker)
b
Dif. methylated CGIs at exons
CGI Meth (Fb < Ker)
CGI Meth (Ker < Fb)
10,000
10
0.01
Ker RPKM (Log10)
0.01
10
10,000
Fb RPKM (Log10)
c
Fb
Genes
10000
8000
6000
4000
2000
0
0
>0-1
>1-10
>10
Non CGI
CGI
Fb. (RPKM)
d
Ker
Genes
10000
8000
6000
4000
2000
0
0
>0-1
>1-10
>10
Non CGI
CGI
Ker. (RPKM)

Figure S17

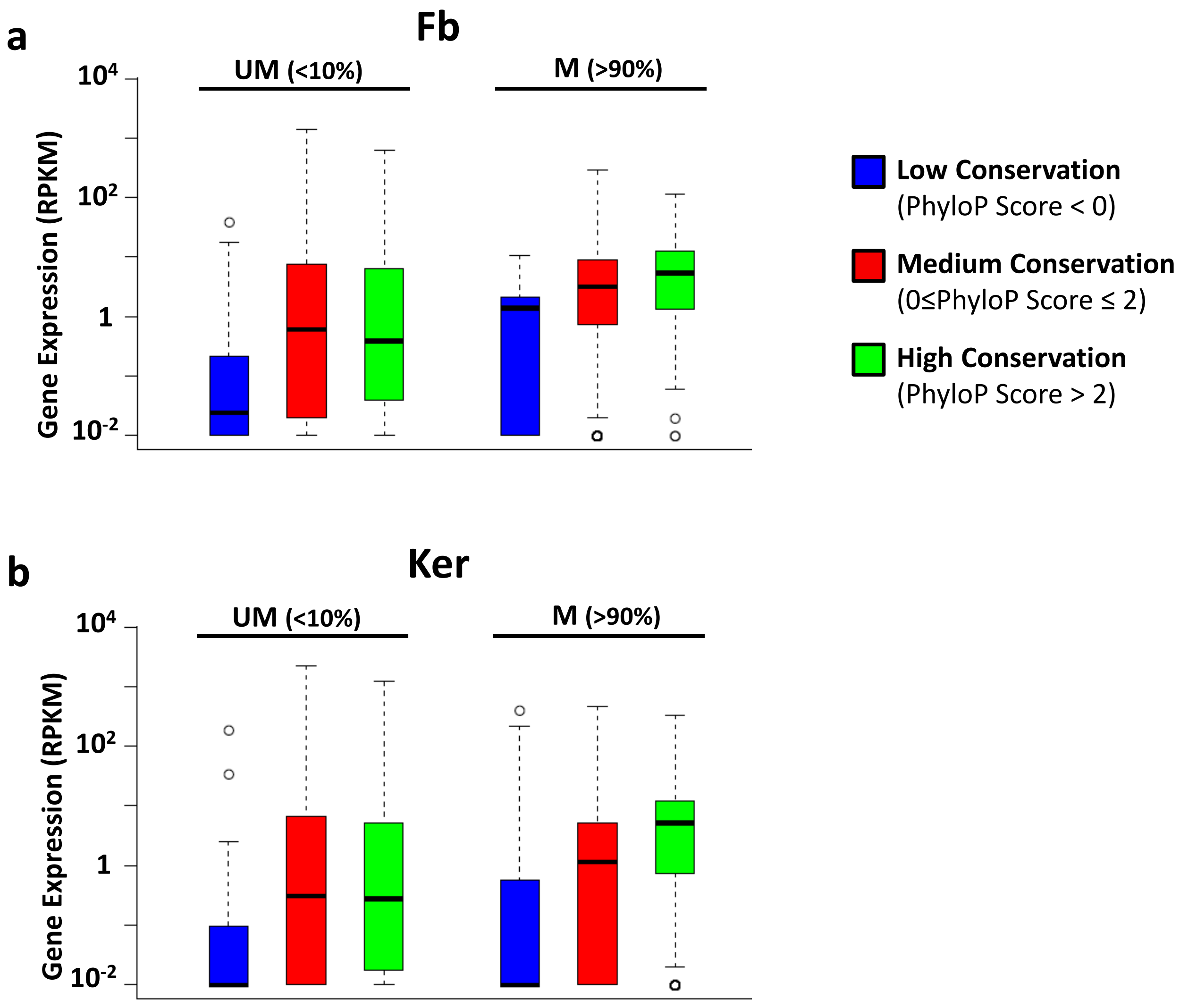
a
Fb
UM (<10%)
M (>90%)
Gene Expression (RPKM)
10^4
10^2
1
10^{-2}
b
Ker
UM (<10%)
M (>90%)
Gene Expression (RPKM)
10^4
10^2
1
10^{-2}
Low Conservation
(PhyloP Score < 0)
Medium Conservation
(0≤PhyloP Score ≤ 2)
High Conservation
(PhyloP Score > 2)

Figure S18

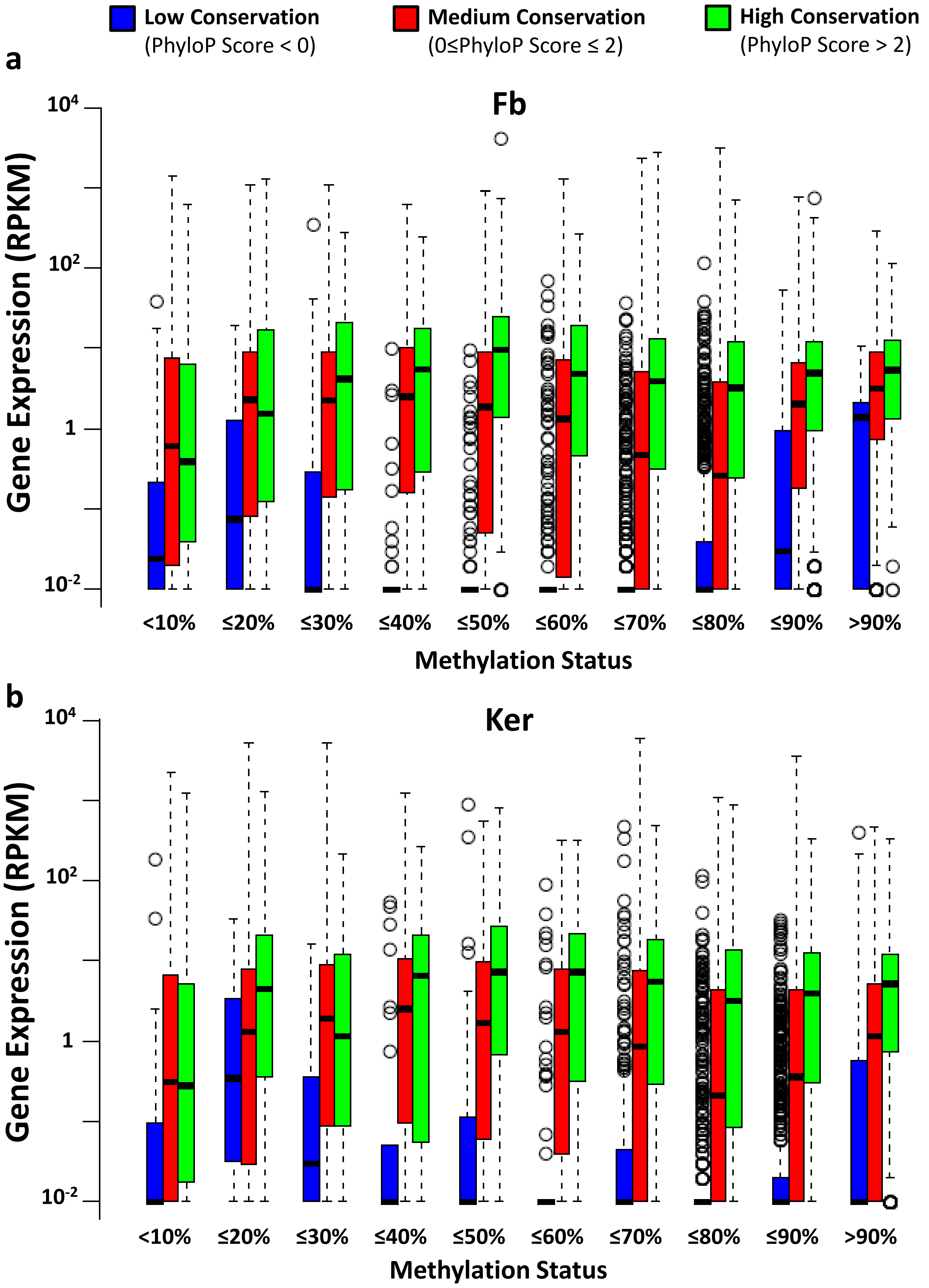
Low Conservation
(PhyloP Score < 0)
Medium Conservation
(0≤PhyloP Score ≤ 2)
High Conservation
(PhyloP Score > 2)
a
Fb
Gene Expression (RPKM)
10^4
10^2
1
10^{-2}
<10%
≤20%
≤30%
≤40%
≤50%
≤60%
≤70%
≤80%
≤90%
>90%
Methylation Status
b
Ker
Gene Expression (RPKM)
10^4
10^2
1
10^{-2}
<10%
≤20%
≤30%
≤40%
≤50%
≤60%
≤70%
≤80%
≤90%
>90%
Methylation Status

Figure S19 RNA-seq signal at the *Fat1* locus

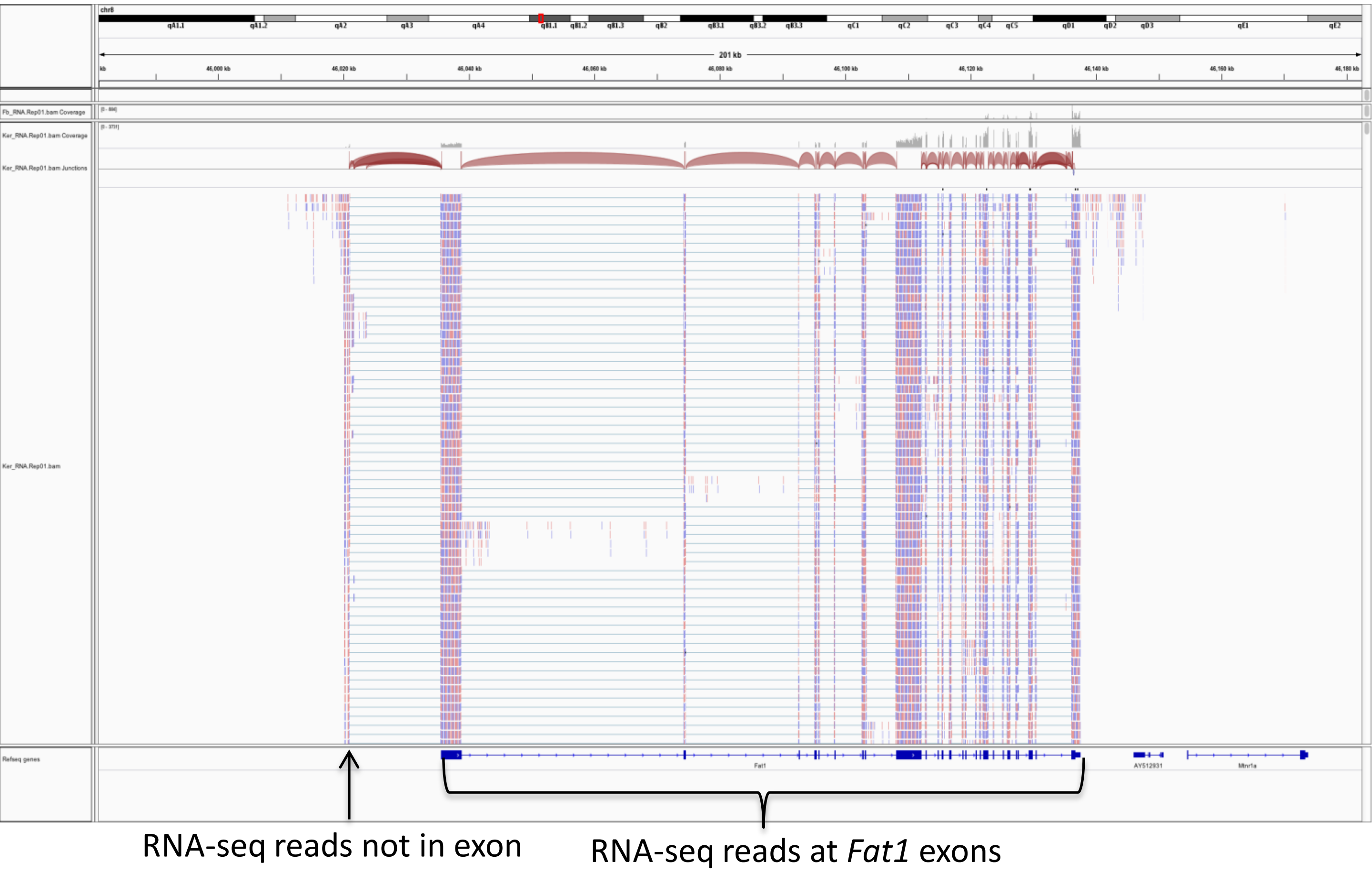

Figure S20

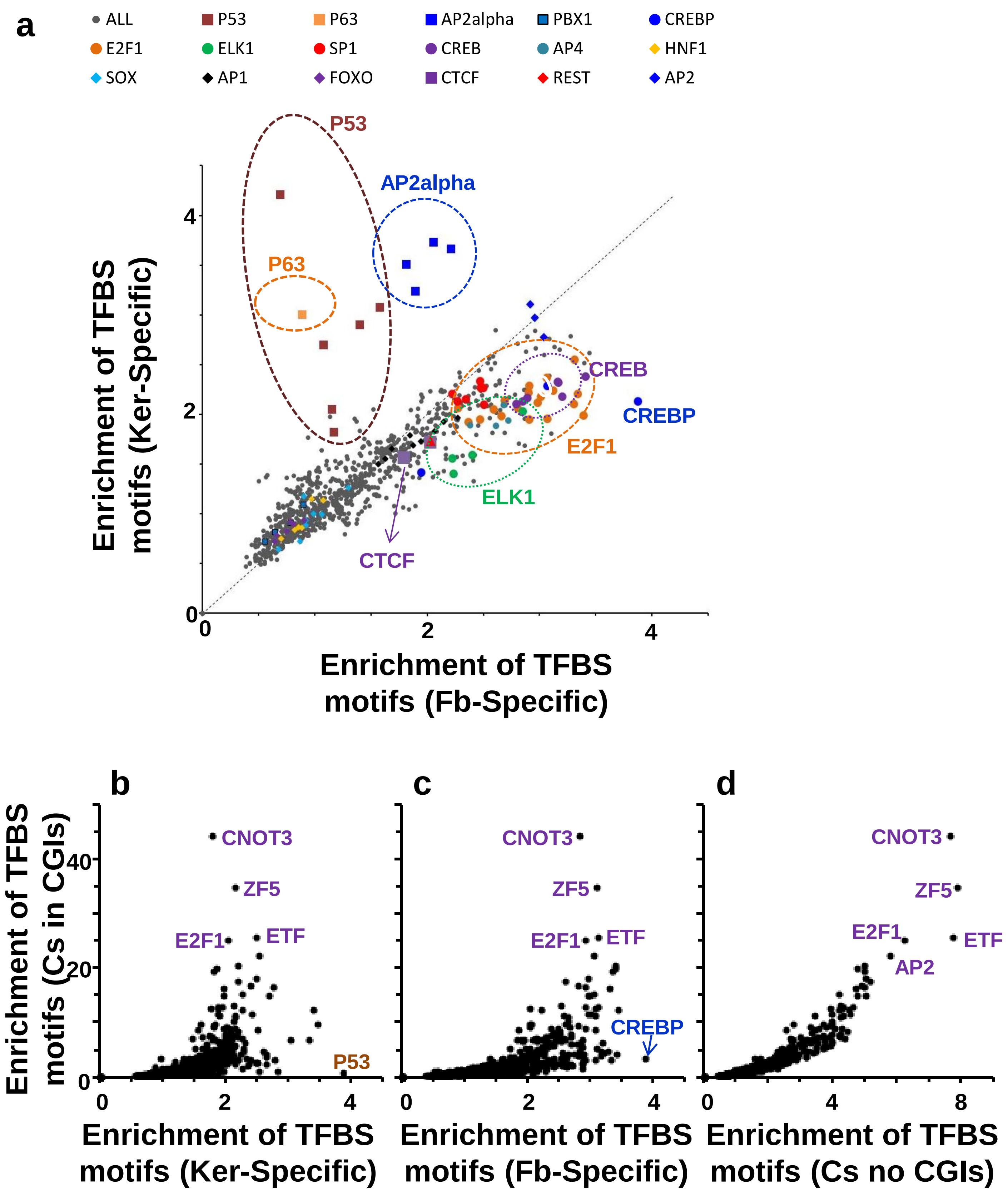
a
ALL
P53
P63
AP2alpha
PBX1
CREBP
E2F1
ELK1
SP1
CREB
AP4
HNF1
SOX
AP1
FOXO
CTCF
REST
AP2
P53
AP2alpha
P63
CREB
CREBP
E2F1
ELK1
CTCF
Enrichment of TFBS motifs (Ker-Specific)
Enrichment of TFBS motifs (Fb-Specific)
0
2
4
b
c
d
CNOT3
ZF5
E2F1
ETF
P53
CREBP
AP2
Enrichment of TFBS motifs (Cs in CGIs)
0
20
40
8
Enrichment of TFBS motifs (Ker-Specific)
Enrichment of TFBS motifs (Fb-Specific)
Enrichment of TFBS motifs (Cs no CGIs)

Figure S21

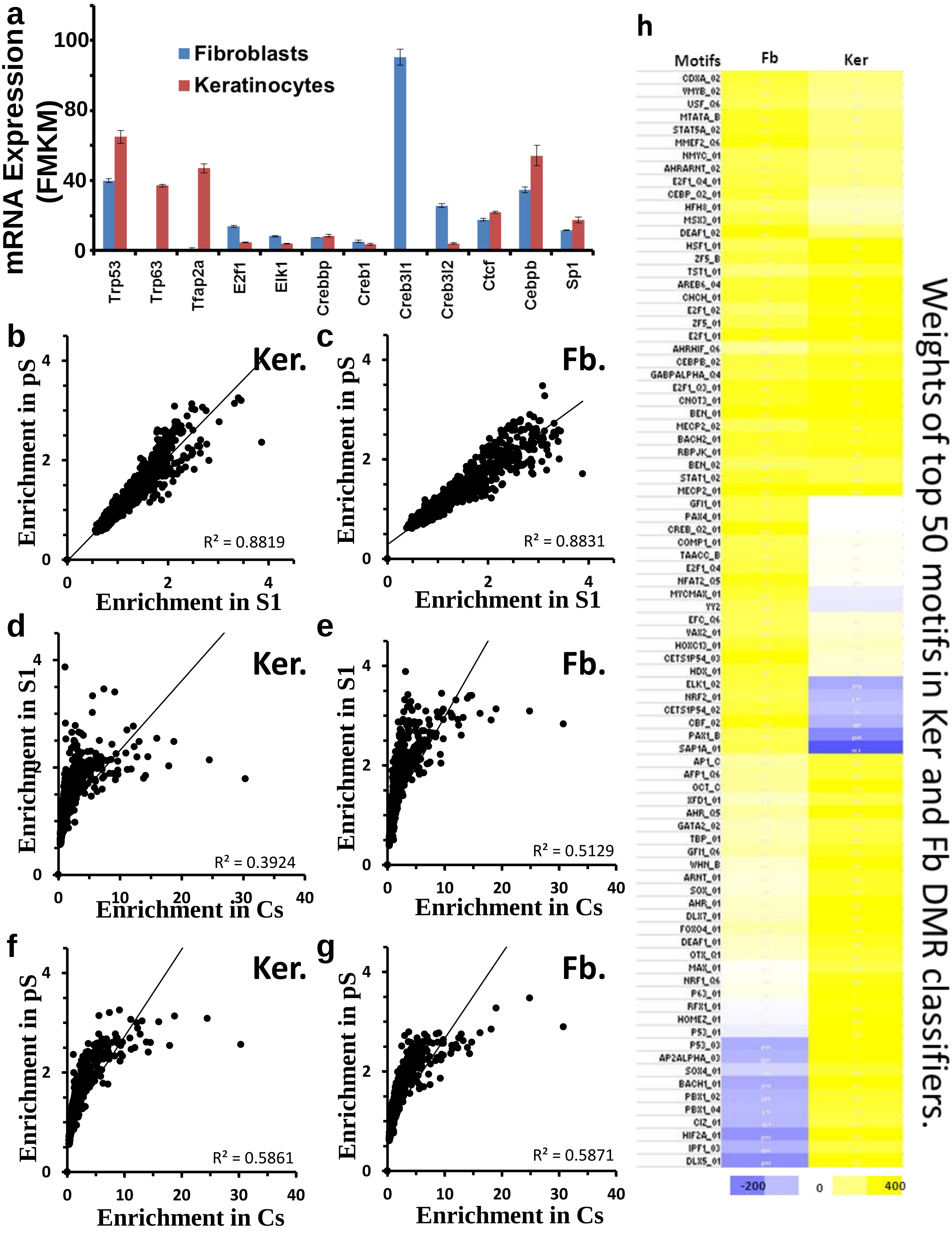

a
mRNA Expression (FMKM)
Fibroblasts
Keratinocytes
Trp53
Trp63
Tfap2a
E2f1
Elk1
Crebbp
Creb1
Creb3l1
Creb3l2
Ctcf
Cebpb
Sp1
b
Ker.
Enrichment in pS
Enrichment in S1
R² = 0.8819
c
Fb.
Enrichment in pS
Enrichment in S1
R² = 0.8831
d
Ker.
Enrichment in S1
Enrichment in Cs
R² = 0.3924
e
Fb.
Enrichment in S1
Enrichment in Cs
R² = 0.5129
f
Ker.
Enrichment in pS
Enrichment in Cs
R² = 0.5861
g
Fb.
Enrichment in pS
Enrichment in Cs
R² = 0.5871
h
Motifs
Fb
Ker
Weights of top 50 motifs in Ker and Fb DMR classifiers.
-200
0
400

Figure S22

a

C/EBPb Peaks

Common (5,034) | **Fb Unique (2,283)** | **Ker Unique (2,645)**

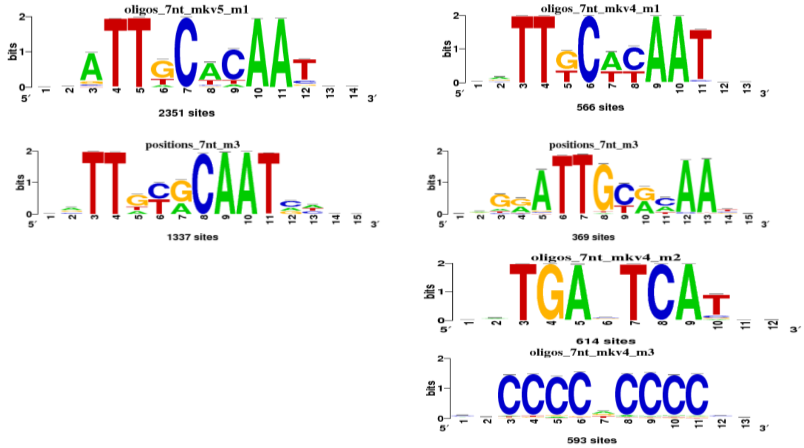

b

CTCF Peaks

Common (9,996) | **Fb Unique (1,671)** | **Ker Unique (5,055)**

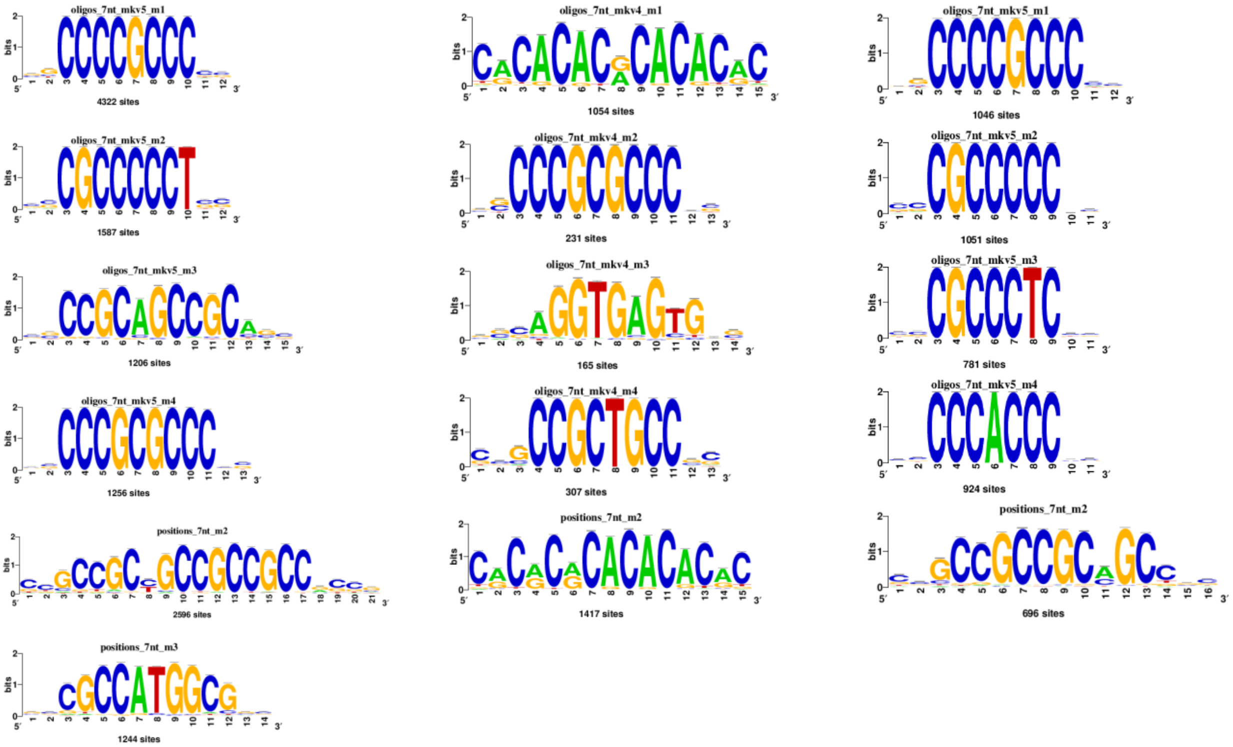

Supplement: Supplementary file 2 — Additional file 2: Figure S1: Methylation of CGs and coverage in fibroblasts and keratinocytes. Figure S2. Adjacent CGs have a similar methylation status. Figure S3. Comparison of CG methylation in different repetitive elements in fibroblasts and keratinocytes. Figure S4. Methylated CGs at exons are more conserved than the unmethylated CGs. Figure S5. HMRs in fibroblasts and keratinocytes. Figure S6. Overlap with CGIs and composition of different methylated CGs in tissues-specific HMRs (S1) and common HMRs (C1-C5). Figure S7. Heatmap of CG methylation for the different groups of keratinocyte HMRs in different cells. Figure S8. Comparison of HMRs in fibroblasts, keratinocytes, embryonic stem cells and neuronal progenitor cells. Figure S9. HMRs are the conserved regions with high GC content as well as CpG density. Figure S10. SINE elements are depleted inside the HMRs, but enriched in the surrounding regions; however, both LTR and LINE elements are depleted in both inside and at the surrounding regions. Figure S11-S12. Comparison of mRNA expression between fibroblasts and keratinocytes. Figure S13. UCSC genome browser screen shots as examples for different types of HMRs. Figure S14. UCSC genome browser screen shots as examples for tissue specific genes expression with TS-HMRs near the TSS of Krt1, Krt5 and NNMT1. Figure S15. UCSC genome browser screen shots as examples for extended HMRs leading to enhanced gene expression of Emilin1, Trp53il1 and Wnt3. Figure S16. Correlation between the methylation difference and gene expression changes in fibroblasts and keratinocytes. Figure S17. Exons with methylated conserved CGs are highly expressed. Figure S18. Exons with methylated conserved CGs are highly expressed. Figure S19. RNA-seq signal at the Fat1 locus. Figure S20. Transcription factor binding sites (TFBS) in HMRs. Figure S21. Comparison of enriched TFBS motifs in the fibroblasts and keratinocyte HMRs. Figure S22. Binding motifs in C/EBPβ and CTCF ChIP-seq peaks. (PDF [file 13072_2014_350_MOESM2_ESM.pdf]
